# Peptidoglycan Association of Murein Lipoprotein Is Required for KpsD-Dependent Group 2 Capsular Polysaccharide Expression and Serum Resistance in a Uropathogenic *Escherichia coli* Isolate

**DOI:** 10.1128/mBio.00603-17

**Published:** 2017-05-23

**Authors:** Jingyu Diao, Catrien Bouwman, Donghong Yan, Jing Kang, Anand K. Katakam, Peter Liu, Homer Pantua, Alexander R. Abbas, Nicholas N. Nickerson, Cary Austin, Mike Reichelt, Wendy Sandoval, Min Xu, Chris Whitfield, Sharookh B. Kapadia

**Affiliations:** a Department of Infectious Diseases, Genentech, South San Francisco, California, USA; b Department of Molecular and Cellular Biology, University of Guelph, Guelph, ON, Canada; c Department of Translational Immunology, Genentech, South San Francisco, California, USA; d Department of Pathology, Genentech, South San Francisco, California, USA; e Department of Protein Chemistry, Genentech, South San Francisco, California, USA; f Department and Bioinformatics, Genentech, South San Francisco, California, USA; Harvard Medical School

**Keywords:** *Escherichia coli*, capsular polysaccharide, cell envelope, complement resistance, murein lipoprotein, polysaccharide export

## Abstract

Murein lipoprotein (Lpp) and peptidoglycan-associated lipoprotein (Pal) are major outer membrane lipoproteins in *Escherichia coli*. Their roles in cell-envelope integrity have been documented in *E. coli* laboratory strains, and while Lpp has been linked to serum resistance *in vitro*, the underlying mechanism has not been established. Here, *lpp* and *pal* mutants of uropathogenic *E. coli* strain CFT073 showed reduced survival in a mouse bacteremia model, but only the *lpp* mutant was sensitive to serum killing *in vitro*. The peptidoglycan-bound Lpp form was specifically required for preventing complement-mediated bacterial lysis *in vitro* and complement-mediated clearance *in vivo*. Compared to the wild-type strain, the *lpp* mutant had impaired K2 capsular polysaccharide production and was unable to respond to exposure to serum by elevating capsular polysaccharide amounts. These properties correlated with altered cellular distribution of KpsD, the predicted outer membrane translocon for “group 2” capsular polysaccharides. We identified a novel Lpp-dependent association between functional KpsD and peptidoglycan, highlighting important interplay between cell envelope components required for resistance to complement-mediated lysis in uropathogenic *E. coli* isolates.

## IMPORTANCE

Uropathogenic *E. coli* (UPEC) isolates represent a significant cause of nosocomial urinary tract and bloodstream infections. Many UPEC isolates are resistant to serum killing. Here, we show that a major cell-envelope lipoprotein (murein lipoprotein) is required for serum resistance *in vitro* and for complement-mediated bacterial clearance *in vivo*. This is mediated, in part, through a novel mechanism by which murein lipoprotein affects the proper assembly of a key component of the machinery involved in production of “group 2” capsules. The absence of murein lipoprotein results in impaired production of the capsule layer, a known participant in complement resistance. These results demonstrate an important role for murein lipoprotein in complex interactions between different outer membrane biogenesis pathways and further highlight the importance of lipoprotein assembly and transport in bacterial pathogenesis.

## INTRODUCTION

Uropathogenic *Escherichia coli* (UPEC) isolates are a significant cause of uncomplicated urinary tract infections (UTIs) (1). UTIs frequently cause bladder infection (cystitis) and can lead to acute kidney infections (pyelonephritis), but they are also a major cause of sepsis, associated with a high mortality rate (2). The cell envelope of the typical Gram-negative bacterium consists of two membranes: a phospholipid inner membrane (IM) and an asymmetrical outer membrane (OM) composed of a phospholipid inner leaflet and an outer leaflet of molecules of lipopolysaccharides (LPS). The IM and OM are separated by the periplasm, which contains the murein (peptidoglycan [PG]) layer. The distinct permeability properties of the outer membrane create a selective permeability barrier allowing access of small nutrients but preventing access of many large and potentially harmful molecules, including certain antibiotics (3, 4). UPEC isolates are typically covered in a “group 2” surface polysaccharide capsule (5, 6) and are major determinants of virulence in *E. coli* isolates causing urinary tract infections, meningitis, and bloodstream infections (reviewed in reference 7). The “group 2” terminology refers to capsules composed of diverse capsular polysaccharide structures (K antigens) with differing antigenic epitopes that are assembled by a conserved strategy (6, 8).

In Gram-negative bacteria, many lipoproteins are important for cellular functions from bacterial cell-envelope homeostasis to cell division and virulence (reviewed in reference 9). While not classically defined as virulence factors, two lipoproteins, Lpp and Pal, are highly expressed in many Gram-negative bacterial species and are important for linking the OM and peptidoglycan layer. Lpp and Pal are both transported to the OM via the Lol-ABCDE system (10) and are anchored to the inner leaflet of the OM (11). Lpp, the first bacterial lipoprotein to be identified, is a small (~8-kDa) lipoprotein which has been demonstrated to play an essential role in membrane integrity and permeability mediated through a covalent linkage between the ε-amino group of the C-terminal lysine residue in Lpp and the meso-diaminopimelic acid residue on the peptidoglycan peptide stem (12–14). Lpp is the most abundant OM protein (OMP) in *E. coli* at ~500,000 molecules per cell. It is found as a PG-bound periplasmic form and in a form that spans the outer membrane to become surface exposed (15). *E. coli* mutants deficient in Lpp exhibit increased OM permeability, leakage of periplasmic components, and increased outer membrane vesicle (OMV) release (16, 17). Pal, like Lpp, is anchored to the inner leaflet of the OM and interacts with OmpA and Lpp and the PG layer (11, 18). Pal is part of the Tol/Pal system, a conserved multiprotein complex embedded in the *E. coli* OM. Pal-deficient mutants exhibit decreased OM integrity, increased susceptibility to antibiotics and detergents, increased leakiness of periplasmic proteins, and increased OMV release (19, 20). To date, most studies on Lpp and Pal have been performed using model laboratory-adapted strains (typically *E. coli* K-12), and thus details of the roles of Lpp and Pal, if any, in *in vivo* infections are still largely unknown. While Phan et al. demonstrated a link between Lpp and serum resistance in a representative of the *E. coli* ST131 lineage, the studies were performed *in vitro* (21). Furthermore, the precise mechanism(s) by which Lpp mediates serum resistance has not been systematically investigated, nor is it known if Pal has a similar role.

The objectives of the present study were to address these gaps in understanding and to test the hypothesis that the altered serum resistance in *lpp* mutants was due to effects on surface polysaccharides, the major contributors to serum resistance. Using *E. coli* CFT073, a UPEC isolate which was isolated from a hospitalized patient with acute pyelonephritis and bacteremia (22), we showed that both Lpp and Pal are crucial for virulence in a mouse bacteremia infection model. However, we demonstrated that they have different roles in pathogenesis, since the *in vivo* role of Pal is independent of any effect on blockade of complement lytic activity. In contrast, Lpp is essential for *in vivo* infection due to its role in preventing complement-mediated bacterial clearance. This activity is due (at least in part) to the role of Lpp in supporting the assembly of group 2 capsular polysaccharide.

## RESULTS

### Lpp is critical for preventing complement-mediated clearance *in vivo* and resistance to complement-mediated lysis *in vitro*.

**FIG 1:**
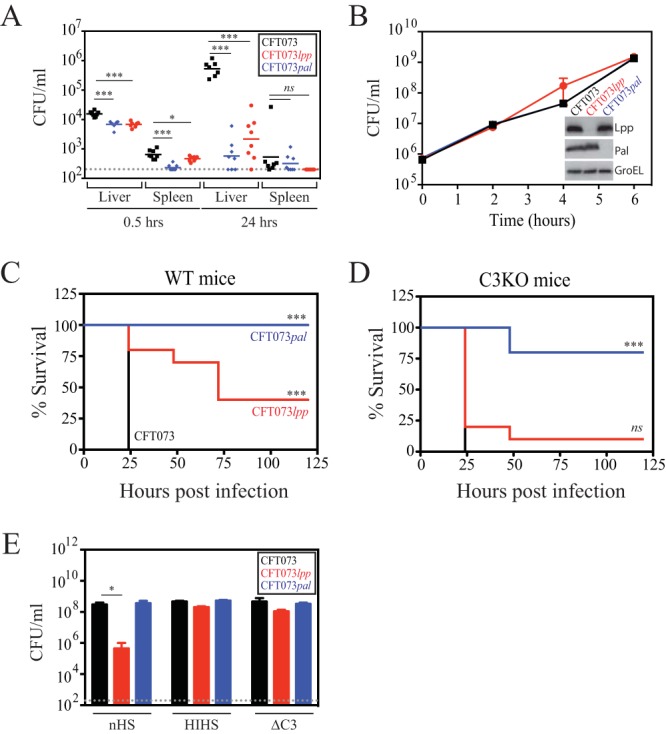
CFT073 *lpp* and CFT073 *pal* are attenuated *in vivo*, but only CFT073 *lpp* shows decreased viability after incubation with human serum *in vitro*. (A) Infection of neutropenic A/J mice with WT CFT073 (black), CFT073 *lpp* (red), and CFT073 *pal* (blue) cells. At 30 min and 24 h postinfection, bacterial burdens in the liver and spleen were enumerated. These data are representative of results from two independent experiments. The overall *P* value for the analysis of variance (ANOVA) is <0.0001. Pairwise comparisons were analyzed using regular unpaired *t* tests (*, *P* = 0.0374; ***, *P* < 0.001) and are denoted in the graphs. (B) Growth of WT CFT073 (black), CFT073 *lpp* (red), and CFT073 *pal* (blue) in LB media. The inset shows an Western immunoblot confirming the lack of Lpp and Pal expression in the CFT073 *lpp* and CFT073 *pal* deletion strains, respectively. Data (means ± SEM) are representative of the results from two replicates. (C and D) Neutropenic C57BL/6 mice (C) or C3-deficient mice (C3KO) (D) were infected with WT CFT073 (black), CFT073 *lpp* (red), or CFT073 *pal* (blue), and survival was monitored daily for up to 5 days. These data are representative of results from two independent experiments, each of which was performed with 8 animals per infection group. *P* values were determined using the log rank (Mantel-Cox) test and were adjusted for multiple testing using Bonferroni correction (*ns*, *P* = 0.1462; CFT073 *lpp*, *P* < 0.001 [***]). (E) WT CFT073 (black), CFT073 *lpp* (red), and CFT073 *pal* (blue) cells were incubated in the presence of normal human serum (nHS), heat-inactivated human serum (HIHS), or C3-depleted serum (ΔC3). *P* values were calculated by regular unpaired *t* tests comparing CFU levels per milliliter to WT UPEC CFU levels per milliliter for each treatment group (*P* = 0.02 [*]). Data (means + SEM) are representative of results from three replicates.

As CFT073 was isolated from a patient with bacteremia (22), a mouse bacteremia model was used to elucidate the roles of Lpp and Pal *in vivo*. Neutropenic A/J mice were infected by intravenous tail vein injection with 5 × 10^5^ CFU of wild-type (WT) CFT073 or isogenic mutants with complete deletions in *lpp* (CFT073 *lpp*) or *pal* (CFT073 *pal*). CFU levels in the liver and spleen were enumerated at 30 min and 24 h postinfection. Compared to WT CFT073, CFT073 *lpp* and CFT073 *pal* were both significantly attenuated at 24 h postinfection, which was reflected in reductions of CFU levels in the liver (~97-fold and ~497-fold, respectively) and the spleen (~20-fold and ~10-fold, respectively) (Fig. 1A). In contrast, CFT073 *lpp* and CFT073 *pal* had no apparent growth defects in culture in LB medium *in vitro* (Fig. 1B).

To investigate the possible role of complement in CFT073 virulence, wild-type C57BL/6 and C3-deficient (C3KO) mice were infected with WT CFT073, CFT073 *lpp*, or CFT073 *pal* and mouse lethality was monitored. If the main role of Lpp or Pal is to prevent complement-mediated bacterial clearance *in vivo*, infection of complement-deficient C3KO mice by CFT073 *lpp* or CFT073 *pal* should rescue the virulence defects in the mutants. As expected from the results described above, CFT073 *lpp* and CFT073 *pal* were both significantly attenuated in wild-type C57BL/6 mice compared to WT CFT073 (Fig. 1C). While levels of CFT073 *pal* remained attenuated in the C3KO mice, survival of CFT073 *lpp* was restored to WT CFT073 levels in the C3KO mice (Fig. 1D). This result clearly demonstrates that Lpp plays a major role in preventing complement-mediated bacterial clearance but that the clearance of CFT073 *pal* clearly involves other host factors.

### Investigation of the role of Lpp in protecting against *in vitro* serum killing.

To investigate the details of complement-dependent *in vivo* attenuation of CFT073 *lpp*, we tested whether CFT073 *lpp* was sensitive to serum killing *in vitro*. We and others have demonstrated that, compared to normal human serum, fresh mouse serum loses significant complement activity *ex vivo*, due to rapid cleavage of C3 (see Fig. S1A in the supplemental material) (23, 24). To overcome this problem, we used human serum for all *in vitro* serum killing experiments. WT CFT073, CFT073 *lpp*, and CFT073 *pal* were incubated with normal human serum (nHS), heat-inactivated human serum (HIHS), or serum that was depleted of factor C3 (ΔC3), which is essential for complement activation. WT CFT073 viability and CFT073 *pal* viability were unaffected by incubation with nHS (Fig. 1E). In contrast, under the same conditions, CFT073 *lpp* exhibited an ~660-fold drop in CFU/ml, consistent with its role in serum resistance and with previous work from others (21). This was confirmed by restoration of bacterial viability when serum complement was inactivated by either heat (HIHS) or C3 depletion (ΔC3) (Fig. 1E). The loss in viability of CFT073 *lpp* after incubation with nHS is likely not explained by a general OM permeability defect under conditions of growth in LB media as there were no significant differences in the sensitivities of CFT073 *lpp* to antibiotics or toxic compounds compared to WT CFT073, with the exception of a modest increase in sensitivity of CFT073 *lpp* to sodium dodecyl sulfate (SDS), as has been previously identified (see Table S1 in the supplemental material). Collectively, these data suggest that while both Lpp and Pal are critical for *in vivo* infection, only Lpp is required for complement resistance of CFT073. As we focused on elucidating the mechanism by which Lpp prevents complement activation and bacterial lysis, we included CFT073 *pal* as a control in key experiments since our data suggest that the attenuation of CFT073 *pal in vivo* is complement independent.

10.1128/mBio.00603-17.2 FIG S1  Confirmation of the lack of classical and alternative pathway complement activity in individual depleted human sera. (A) *In vitro* complement activity of fresh mouse serum compared to normal human serum (nHS) or heat-inactivated human serum (HIHS). *P* values were calculated using unpaired *t* tests (**, *P* = 0.0077; ***, *P* < 0.001). (B and C) The ability of human sera depleted of complement factors to activate alternative and classical complement pathways *in vitro*. The sera were tested for their ability to lyse antibody-coated sheep erythrocytes (to measure classical complement pathway activation) and to lyse nonopsonized rabbit erythrocytes (to measure alternative complement pathway activation). Assays were performed per the recommendations of the manufacturer (Complement Technology). Data are representative of results of at least two independent experiments, with each performed in duplicate. *P* values were calculated using unpaired *t* tests (**, *P* < 0.01; ***, *P* < 0.001). Download FIG S1, EPS file, 1 MB. Copyright © 2017 Diao et al. 2017 Diao et al. This content is distributed under the terms of the Creative Commons Attribution 4.0 International license.

10.1128/mBio.00603-17.5 TABLE S1  MICs of known antibacterials and other compounds against WT CFT073, CFT073 *lpp*, and CFT073 *pal* strains. Download TABLE S1, DOC file, 0.1 MB. Copyright © 2017 Diao et al. 2017 Diao et al. This content is distributed under the terms of the Creative Commons Attribution 4.0 International license.

We further confirmed that Lpp inhibits the classical and alternative complement activation pathways by testing viability of WT CFT073 and CFT073 *lpp* after incubation with human sera depleted of individual components critical for activation of these pathways. Human sera depleted for complement factors essential for activation of classical pathway (C1q and C4), alternative pathway [Factor B (FB) and Factor D (FD)], terminal complement complex (TCC) (C5 and C9), or both pathways (C3), were unable to activate the expected complement pathways (Fig. S1B and S1C). While depletion of complement components had a minimal effect on viable counts of WT CFT073, as anticipated (Fig. S2A), depletion of factors essential for complement activation by the classical pathway or the alternative pathway resulted in CFT073 *lpp* viable counts equivalent to those observed with WT CFT073 (Fig. S2B). Depletion of complement C5 and C9, components of the TCC, resulted in a similar loss of viability. Addition of recombinant factors to the corresponding depleted sera restored serum sensitivity in CFT073 *lpp* (Fig. S2B). This was further corroborated by the increased deposition of C3, C5, C7, and C9 complement components on CFT073 *lpp* cells compared to WT CFT073 measured by flow cytometry (Fig. S2C). Flow cytometry experiments also confirmed that the sera used in the serum resistance assays contained human immunoglobulins (IgG and IgM) that bound to WT CFT073 and CFT073 *lpp* cells (Fig. S2D), suggesting a mechanism for activation of the classical complement pathway on CFT073. Taken together, these data demonstrate that Lpp is essential for protecting CFT073 against both classical and alternative complement pathway-mediated bacteriolysis *in vitro*. Some bacteria, such as *Neisseria meningiditis*, can evade complement-mediated lysis by binding and increasing the local concentration of negative complement regulators such as factor H (25). However, no role for factor H could be detected in CFT073 serum resistance (Fig. S2E), despite a significant increase in activation of the alternative complement pathway in the factor H-depleted serum (Fig. S1C).

10.1128/mBio.00603-17.3 FIG S2  Lpp inhibits both classical and alternative complement pathways. (A and B) WT CFT073 (A) and CFT073 *lpp* (B) bacteria were incubated with commercially available human sera depleted for components required for activation of the classical complement pathways (C1q, C4), the alternative complement pathways (factor B/FB, factor D/FD) or the terminal complement complex pathways (C5 and C9). Individual recombinant complement components were added back (denoted by “+”) to their respective depleted sera to reconstitute complement-active serum per the recommendations of the manufacturers. These data are representative of results of at least two independent experiments. *P* values were calculated using unpaired *t* test (*, *P* < 0.05; ***, *P* < 0.001) and are denoted in the graphs. (C) Deposition of complement components C3, C5, C7, and C9 (detected as C9 neoantigen, which exists in C9 only when part of the TCC) on WT CFT073 (black histograms) and CFT073 *lpp* (red histograms). Bacterial cells incubated with nHS were either left unstained (gray, filled histograms) or stained with antibodies against C3, C5, C7, or C9neo and analyzed by flow cytometry. As controls, bacterial cells incubated with human sera depleted of C3, C5, C7, and C9 are indicated by the dotted histogram. (D) Deposition of human IgG/IgM on WT CFT073 (black histograms), and CFT073 *lpp* (red histograms) cells. Unstained cells are denoted by gray, filled histograms. Data from panels C and D are representative of results of at least four independent experiments. (E) Depletion of factor H does not result in enhanced bacterial lysis of WT CFT073. Data are representative of results of at least two independent experiments, with each performed in duplicate. Download FIG S2, EPS file, 1.7 MB. Copyright © 2017 Diao et al. 2017 Diao et al. This content is distributed under the terms of the Creative Commons Attribution 4.0 International license.

### Lpp is critical for serotype K2 capsular polysaccharide expression in CFT073.

**FIG 2:**
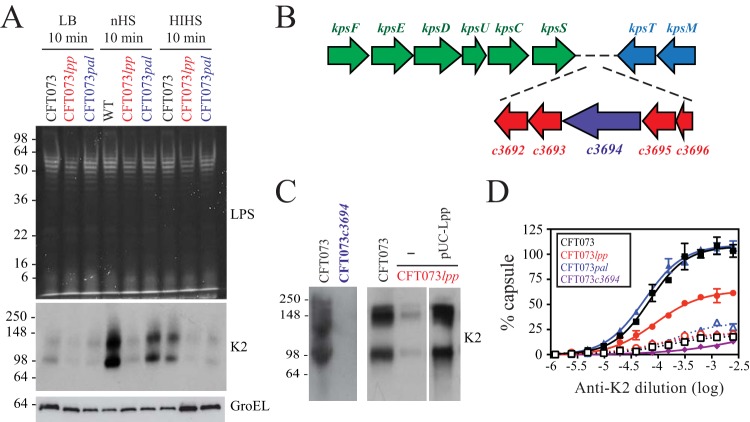
Decreased K2 capsular polysaccharide in CFT073 *lpp*. (A) WT CFT073, CFT073 *lpp*, and CFT073 *pal* were incubated with nHS, HIHS, or LB media for 10 min, and the amounts of LPS (top panel, Pro-Q emerald 300 LPS gel stain) and K2 capsular polysaccharide (middle panel, Western immunoblot using anti-K2 capsular polysaccharide polyclonal antibody) were determined. CFT073 *pal* was included as a control as initial data (Fig. 1) suggested that CFT073 *pal* is not sensitive to complement-mediated lysis and that its attenuation *in vivo* is complement independent. Samples were normalized based on equivalent protein concentrations prior to loading, and loading was confirmed by Western blotting analyses using an anti-GroEL antibody (bottom panel). These data are representative of results of at least three independent experiments. (B) Organization of the genetic loci required for group 2 capsular polysaccharide expression in CFT073. Region 1 (green) and region 3 (blue), which contain genes that are conserved across the group 2 capsule serotypes, flank K2 serotype-specific region 2. c*3694* (purple) was selected for mutation to eliminate K2 capsular polysaccharide biosynthesis. (C) Western immunoblot analysis demonstrating specificity of anti-K2 antibody and Lpp-specific decreased capsular polysaccharide levels in CFT073 *lpp*. WT CFT073 and CFT073 *c3694* total cell lysates were separated by SDS-PAGE and probed with anti-K2 antibody, confirming the lack of K2 capsular polysaccharide production by CFT073 *c3694* cells. WT CFT073 and CFT073 *lpp* total cell lysates were separated by SDS-PAGE and probed with anti-K2 antibody, confirming that the lack of K2 capsular polysaccharide production by CFT073 *lpp* cells is due to Lpp deletion. (D) Whole-cell ELISA measuring the surface-localized K2 capsular polysaccharides on WT CFT073 (black), CFT073 *lpp* (red), and CFT073 *pal* (blue) cells. ELISA plates were coated with intact WT or mutant cells grown in LB media and incubated with anti-K2 (solid symbols and curves) or anti-K12 (a different capsule serotype; open symbols and dotted curves) rabbit polyclonal antibodies. As expected, CFT073 *c3694* cells showed no reactivity with either antibody (purple curves). OD_450_ values were normalized to WT CFT073 values and curve fitted using nonlinear regression analysis. Data (means ± SEM) are representative of results from two replicates.

Since *lpp* mutants have pleiotropic phenotypes, Lpp might act directly in maintaining serum resistance, or its effect might be mediated indirectly and might involve other cellular components. Due to the pivotal importance of cell surface polysaccharides in serum resistance in *E. coli* (7), we examined the levels of LPS O antigen and capsular polysaccharides. WT CFT073, CFT073 *lpp*, and CFT073 *pal* were incubated in nHS, HIHS, or LB growth media for 10 min, a time point at which there was minimal reduction in cell viability (data not shown), and the levels of LPS O-antigen and capsular polysaccharides were analyzed. As mentioned previously, CFT073 *pal* was included as a control since previous data (Fig. 1) suggest that CFT073 *pal* is not sensitive to complement-dependent serum killing. No obvious differences were observed in O-antigen expression levels (based on the sodium dodecyl sulfate-polyacrylamide gel electrophoresis [SDS-PAGE] profiles of O-antigen-substituted smooth LPS molecules) in either CFT073 *lpp* or CFT073 *pal* compared to WT CFT073 under all treatment conditions (Fig. 2A). Western immunoblot analyses using an antibody against the conserved LPS core also revealed no changes in the overall amount of LPS (data not shown). To probe cell-associated capsular polysaccharide, we used an anti-K2 antibody; the serotype of CFT073 is O6:K2:H1 (26). Western immunoblots revealed that the amount of K2 capsular polysaccharide in CFT073 *lpp* cells grown in LB medium was decreased relative to WT and CFT073 *pal* cells (Fig. 2A, middle panel). This difference was more pronounced after treatment with nHS, relative to the corresponding LB-grown control cells (Fig. 2A). Group 2 capsular polysaccharide production is subject to complex transcriptional regulation (6), and 10 min of incubation in nHS is sufficient for *de novo* synthesis of additional capsular polysaccharide assembly on the cell surface (27). The induction of K2 capsular polysaccharide by nHS was presumed to be dependent in part on complement deposition as the induction was decreased when cells were incubated with HIHS (Fig. 2A). Polyclonal antiserum against another K antigen (serotype K12) was used as a negative control, and the results showed no cross-reactivity with the bands detected using the anti-K2 antisera (data not shown). To confirm that the molecular species detected as shown in Fig. 2A were in fact specific to K2 capsular polysaccharides, the *c3694* gene was deleted in CFT073 (CFT073 *c3694*). *c3694* is located in the *kps* (capsule synthesis and export) locus and encodes a predicted bifunctional protein containing a glycerophosphate transferase and a GT1-family glycosyltransferase, functions consistent with the K2 capsular polysaccharide structure (unpublished data and Fig. 2B). Lack of K2 reactivity in CFT073 *c3694* using the anti-K2 polyclonal antibody confirmed the specificity of K2 capsule detection by SDS-PAGE (Fig. 2C).

**FIG 3:**
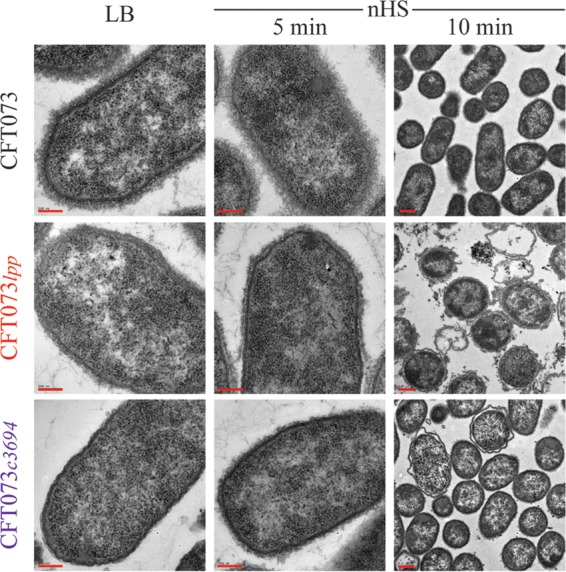
Decreased K2 capsular polysaccharide expression in CFT073 *lpp* revealed by electron microscopy. WT CFT073 or CFT073 *lpp* was treated with LB medium or nHS for up to 10 min, incubated with anti-K2 antibody to stabilize the capsular polysaccharides, and stained with ruthenium Red. Bacterial cells treated with HIHS were indistinguishable from those treated with LB media (data not shown). For the images representing results of LB and nHS treatment (5 min), bar = 200 nm; for the images representing results of nHS treatment (10 min), bar = 0.5 μM.

Western immunoblots detect total cellular K2 capsular polysaccharide but cannot distinguish glycans accumulating inside the cell from those assembled on the cell surface. Therefore, we performed enzyme-linked immunosorbent assays (ELISA) by measuring the amount of anti-K2 antibody reactivity to whole cells. Our results confirmed the substantial decrease in amounts of surface K2 capsular polysaccharide in CFT073 *lpp* cells relative to either WT CFT073 or CFT073 *pal* cells (Fig. 2D). The change in capsule assembly in CFT073 *lpp* was also visualized using an electron microscope (EM). WT CFT073, CFT073 *lpp*, and CFT073 *c3694* were incubated with anti-K2 capsule antibodies to stabilize the capsule prior to processing for electron microscopy. The cellular morphologies of WT and mutant bacteria were comparable under conditions of growth in LB medium (Fig. 3 and data not shown). Capsular polysaccharides were evident on the surface of WT CFT073 grown in LB media, and more-extensive surface glycans were observed after incubation in nHS (Fig. 3). CFT073 *lpp* cells showed less capsule density than WT CFT073 cells, with no obvious increase after exposure to nHS (Fig. 3). In addition, CFT073 *lpp* cells incubated with nHS showed a loss in membrane integrity and loss of cytoplasmic contents; this effect was absent under conditions of incubation with HIHS (data not shown). Interestingly, CFT073 *c3694* cells showed lower levels of membrane perturbations and nucleoid condensation at the 10-min time point, suggesting that the complement sensitivity of CFT073 *lpp* is complex and is explained only partially by loss of K2 capsule. Collectively, these data demonstrate that the levels of capsular polysaccharides and the amount of cell surface capsular structure are reduced in the absence of Lpp and that *lpp* mutants are unable to increase capsule production in response to serum factors.

### Elevated capsular polysaccharide expression is dependent on the linkage of Lpp to peptidoglycan.

**FIG 4:**
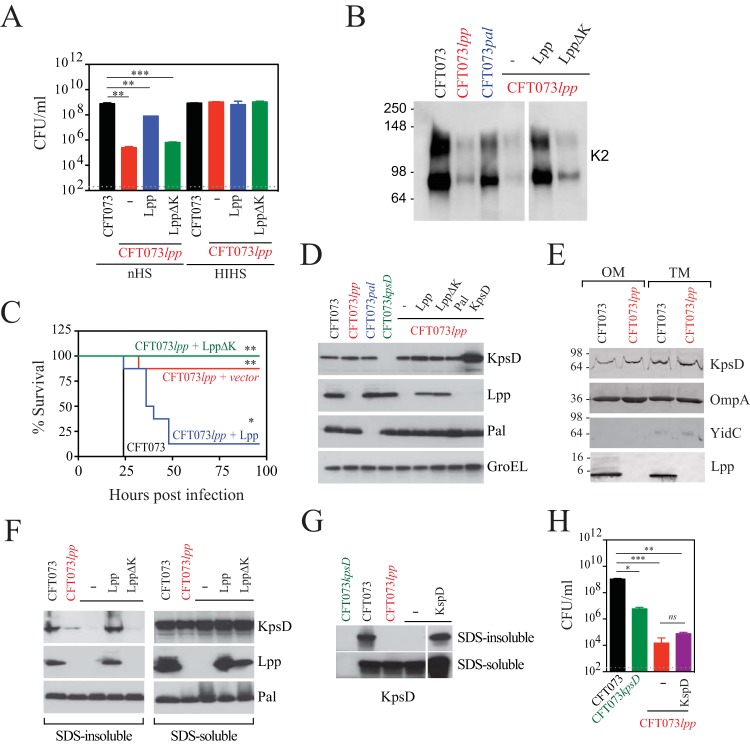
Full-length PG-associated Lpp, but not an Lpp variant with a deletion of the C-terminal lysine, can rescue CFT073 *lpp* defects in serum resistance, capsular polysaccharide expression, and virulence in mice. CFT073 *lpp* was transformed with an empty plasmid (pUC, red) or a plasmid encoding full-length Lpp (pUC-Lpp, blue) or a plasmid encoding Lpp lacking the C-terminal lysine (pUC-LppΔK, green). (A to C) Plasmid-carrying strains were compared to WT CFT073 (black) for their resistance to serum killing (A), production of K2 capsular polysaccharides (B), and virulence *in vivo* (C). For the serum killing experiments represented in panel A, the overall *P* value for the ANOVA is <0.0001. Pairwise comparisons were analyzed using regular unpaired *t* tests (for CFT073 *lpp* + pUC, *P* = 0.0013 [**]; for CFT073 *lpp* + pUC-Lpp, *P* = 0.0083 [**]; for CFT073 *lpp* + pUC-LppΔK, *P* = 0.0009 [***]) and are denoted in the graphs. For the mouse lethality studies represented in panel C, *P* values were determined using the log rank (Mantel-Cox) test and were adjusted for multiple testing using Bonferroni correction (for CFT073 *lpp* + pUC, *P* = 0.0039 [**]; for CFT073 *lpp* + pUC-Lpp, *P* = 0.0249 [*]; for CFT073 *lpp* + pUC-LppΔK, *P* = 0.0027 [**]). (D) Total cell lysates of WT CFT073, CFT073 *lpp*, CFT073 *kpsD*, CFT073 *pal*, or CFT073 *lpp* complemented with empty pUC19 plasmid (-) or pUC19 expressing Lpp, LppΔK, or KpsD were separated by SDS-PAGE and probed with anti-KpsD, anti-Lpp, and anti-Pal antibodies. Anti-GroEL antibody was used as a loading control. (E) Outer membrane (OM) and total membrane (TM) preparations from WT CFT073 and CFT073 *lpp* were separated by SDS-PAGE and probed with anti-KpsD, anti-OmpA, anti-YidC, and anti-Lpp antibodies. (F) Full-length Lpp but not LppΔK could rescue levels of PG-associated KpsD in CFT073 *lpp*. SDS-insoluble (peptidoglycan-associated) and SDS-soluble (OM/IM-associated) protein fractions were isolated from WT CFT073 and CFT073 *lpp* strains complemented with plasmids carrying the appropriate genes as described above. (G and H) KpsD overexpression in CFT073 *lpp* rescues levels of PG-associated KpsD form but not CFT073 *lpp* serum resistance. (G) SDS-insoluble (peptidoglycan-associated) and SDS-soluble (OM/IM/cytoplasm-associated) protein fractions were isolated from WT CFT073 and CFT073 *lpp* complemented with vectors as described above. (H) WT CFT073 (black), CFT073 *kpsD*, and CFT073 *lpp* transformed with an empty vector pUC (red) or a vector encoding KpsD (purple) were incubated with nHS. No loss in viability for any strain was detected upon incubation with HIHS (data not shown). Data (means ± SEM) are representative of results from two replicates. The overall *P* value for the ANOVA is 0.0004. Pairwise comparisons were analyzed using regular unpaired *t* tests (for CFT073 *kpsD*, *P* = 0.0164 [**]; for CFT073 *lpp* + pUC, *P* = 0.0007 [***]; for CFT073 *lpp* + pUC − KpsD, *P* = 0.0059 [**]) and are denoted in the graphs. No difference was observed between CFT073 *lpp* complemented with empty vector and CFT073 *lpp* complemented with vector expressing KpsD (*ns*, *P* = 0.353).

While a fraction of Lpp expressed in *E. coli* is covalently cross-linked to peptidoglycan (PG) through its C-terminal lysine, the majority (almost two-thirds) of Lpp is free (28). To determine whether the PG-associated fraction of Lpp is specifically required for K2 capsule production and serum resistance, an Lpp (LppΔK) variant lacking the C-terminal lysine (required for linkage to PG) was tested for its ability to rescue resistance to *in vitro* serum killing in CFT073 *lpp*. While complementation with a plasmid expressing wild-type Lpp almost completely rescued CFT073 *lpp* serum resistance, complementation with LppΔK did not (Fig. 4A). These results correlated with both the amounts of K2 capsular polysaccharide produced (Fig. 4B) and the levels of virulence in mice (Fig. 4C). The expression levels of wild-type Lpp and LppΔK proteins were comparable as measured by Western immunoblot analysis of total cell lysates (Fig. 4D). Taken together, these results suggest that the PG cross-linked form of Lpp is important for both proper capsular polysaccharide assembly and virulence.

### Proteomic analysis of CFT073 *lpp* reveals changes in levels of multiple membrane-associated proteins.

A proteomics approach was used to determine whether expression of membrane-embedded/membrane-associated proteins involved in capsular polysaccharide assembly was altered in CFT073 *lpp* cells. WT CFT073 or CFT073 *lpp* cells were incubated with LB media, nHS, or HIHS for 10 min. Total membranes (TM) were isolated, and samples were analyzed by mass spectrometry (MS). Based on total spectral counts, levels of 14 proteins decreased and levels of 20 proteins qualitatively increased in abundance in CFT073 *lpp* membrane samples compared to WT CFT073 by more than 2-fold (Tables S2 and S3). As expected, levels of Lpp were detected at substantially lower levels in CFT073 *lpp* than in WT CFT073; the residual signal likely reflects background in this procedure. Importantly, the amount of KpsD appeared lower in CFT073 *lpp* membrane preparations derived from cells incubated in LB medium than in the WT CFT073 membrane preparations (13 versus 47 total spectral matches, respectively; Table S2). KpsD is the putative outer membrane translocon for export of group 2 capsular polysaccharides (8). This apparent reduction did not extend to other components of the translocation machinery as the amounts of KpsE and KpsM showed no obvious changes (Table S4). KpsE is embedded in the IM and possesses a large periplasmic domain, which is thought to interact with KpsD. KpsM is the integral membrane channel component of the ABC transporter that helps define group 2 capsule assembly pathways (8).

10.1128/mBio.00603-17.6 TABLE S2  Levels of membrane-associated bacterial proteins decreased in CFT073 *lpp* versus WT CFT073 identified through LC/MS/MS. Download TABLE S2, DOCX file, 0.1 MB. Copyright © 2017 Diao et al. 2017 Diao et al. This content is distributed under the terms of the Creative Commons Attribution 4.0 International license.

10.1128/mBio.00603-17.7 TABLE S3  Levels of membrane-associated bacterial proteins increased in CFT073 *lpp* versus WT CFT073 identified through LC/MS/MS. Download TABLE S3, DOCX file, 0.1 MB. Copyright © 2017 Diao et al. 2017 Diao et al. This content is distributed under the terms of the Creative Commons Attribution 4.0 International license.

10.1128/mBio.00603-17.8 TABLE S4  Membrane-associated bacterial proteins expressed at similar levels in CFT073 *lpp* versus WT CFT073 identified through LC/MS/MS. Download TABLE S4, DOCX file, 0.4 MB. Copyright © 2017 Diao et al. 2017 Diao et al. This content is distributed under the terms of the Creative Commons Attribution 4.0 International license.

Levels of other cell envelope proteins important for virulence were also lower in CFT073 *lpp* membranes. These included proteins crucial for adhesion to extracellular matrix (ECM) proteins (UpaB), pilus biogenesis (FimD), and UPEC pathogenesis (UpaD) (Table S2). Of the proteins with higher expression levels in CFT073 *lpp* cells, many were involved in cell metabolism (LldD, DlD, GlpD, and GlpT), membrane protein biogenesis and insertion (FfH, FtsY, and SurA), and synthesis of membrane-derived oligosaccharides (MdoB and MdoH) (Table S3). Expression levels of other proteins belonging to major OM assembly pathways, such as those involved in OM insertion of β-barrel proteins and LPS localization to the outer membrane (the Bam and Lpt pathways, respectively) and other major *E. coli* OMPs (LamB, OmpA, OmpF, OmpC, OmpX, and OmpT) were present at comparable levels in WT CFT073 and CFT073 *lpp* cells (Table S4). Multiple complement components were also detected in CFT073 *lpp* membrane samples following incubation with nHS but not with HIHS, adding further support for the idea of increased complement activation on CFT073 *lpp* cells (Table S5). In summary, the proteomic analysis of the membrane composition in CFT073 *lpp* cells identified membrane proteins and pathways that might be affected, directly or indirectly, by the presence of Lpp and which could also contribute to pathogenesis.

10.1128/mBio.00603-17.9 TABLE S5  Human proteins associated with WT CFT073 and CFT073 *lpp* total membranes identified through LC/MS/MS. Download TABLE S5, DOCX file, 0.1 MB. Copyright © 2017 Diao et al. 2017 Diao et al. This content is distributed under the terms of the Creative Commons Attribution 4.0 International license.

### The PG-bound form of Lpp is critical for peptidoglycan association of KpsD and capsular polysaccharide expression.

Previous work by others has established the importance of the K2 capsule in resistance to serum killing by examining the phenotype of a mutant deleted in the so-called “region 2” genes responsible for biosynthesis of the K2 repeat-unit glycan (i.e., c3692 to c3696; Fig. 2B) (26) or of a “region 1” *kpsC* mutant (29) unable to initiate K2 production (30). KpsD, identified in our proteomic analysis described above, is required for capsular polysaccharide export, and it is proposed to form an outer membrane channel to facilitate passage of the polymer to the cell surface (31). A deletion strain (CFT073 *kpsD*) was constructed to confirm its requirement for serum resistance. Export-deficient strains (such as *kpsD* mutants) can still synthesize capsular polysaccharide but retain it within the cell (32). Similar levels of K2 capsular polysaccharide expression were detected in lysates of CFT073 *lpp* and CFT073 *kpsD* cells (Fig. S3A). Deletion of KpsD resulted in serum killing (Fig. S3B), albeit not at the level seen with CFT073 *lpp*. This is consistent with our electron microscopy results (Fig. 3), demonstrating that Lpp mediates both capsule-dependent and capsule-independent mechanisms to resist complement.

10.1128/mBio.00603-17.4 FIG S3  Decreased serum resistance and capsular polysaccharide levels in CFT073 *lpp* and CFT073 *kpsD*. (A) The level of serotype K2 capsular polysaccharide is decreased in CFT073 *lpp* and CFT073 *kpsD* cells. WT CFT073, CFT073 *lpp*, and CFT073 *kpsD* cells were incubated with nHS for 10 min, and the level of K2 capsular polysaccharide was determined by Western immunoblotting using anti-K2 capsular polysaccharide polyclonal antibody. (B) WT CFT073 (black), CFT073 *lpp* (red), and CFT073 *kpsD* (blue) cells were incubated in the presence of normal human serum (nHS) at a final concentration of 100% for 30 min at 37°C. *P* values were calculated comparing CFU levels per milliliter for each treatment group to WT UPEC CFU levels per milliliter. The overall *P* value for the ANOVA is 0.0007. Pairwise comparisons were analyzed using regular unpaired *t* tests (for CFT073 *lpp*, *P* = 0.0035 [**]; for CFT073 *kpsD*, *P* = 0.0259 [*]) and are denoted in the graphs. Data (means + SEM) are representative of results from two replicates. (C) K2 capsular polysaccharide expression in WT CFT073, CFT073 *kpsD*, CFT073 *lpp*, and CFT073 *lpp* complemented with empty plasmid (-), KpsD, full-length Lpp, or Lpp lacking the C-terminal lysine (LppΔK). Download FIG S3, PDF file, 1.1 MB. Copyright © 2017 Diao et al. 2017 Diao et al. This content is distributed under the terms of the Creative Commons Attribution 4.0 International license.

To further investigate the levels of KpsD in WT CFT073 and CFT073 *lpp*, the amounts of KpsD were probed in Western immunoblots using a polyclonal antibody directed against KpsD. Unexpectedly, total KpsD levels in the two strains in total cell lysates (Fig. 4D) or in OM and total membrane (TM) preparations (Fig. 4E) were comparable. This was a surprising result given the observed differences seen in our proteomics experiment. On the basis of our observation that PG-bound Lpp is responsible for serum resistance (Fig. 4A), we asked whether there are differences in KpsD amounts in the PG-enriched SDS-insoluble cellular fraction. The amounts of KpsD in the PG-free SDS-soluble fraction were indistinguishable between WT CFT073 and CFT073 *lpp* (Fig. 4F), consistent with previous reports indicating that large amounts of KpsD are found in a periplasmic form (33). However, the KpsD levels in the SDS-insoluble fraction of CFT073 *lpp* cells were substantially decreased relative to WT CFT073 cell results. Wild-type amounts could be restored by expressing plasmid-encoded Lpp but not LppΔK (Fig. 4F). There was no cross contamination during isolations of the SDS-insoluble and SDS-soluble fractions because the LppΔK protein (which, as shown above, is produced in total amounts comparable to those seen with native Lpp) was confined to the SDS-soluble fraction (Fig. 4F). Overexpression of KpsD from a multicopy plasmid resulted in elevated amounts of KpsD in total cell lysates (Fig. 4D) as well as in SDS-insoluble fractions (Fig. 4G) from CFT073 *lpp* cells. However, this did not restore serum resistance (Fig. 4H) or K2 capsular polysaccharide production (Fig. S3C). We conclude that the inability to restore serum resistance is due to the critical requirement for PG association of KpsD in a strictly Lpp-dependent manner.

## DISCUSSION

Lpp was discovered approximately 40 years ago and is one of the best-studied bacterial lipoproteins. It exists in both a free form and a PG-bound form (12, 13, 34). Bound-form Lpp trimers maintain the integrity of the bacterial cell surface by covalent interaction between the C-terminal lysine and a meso-diaminopimelic acid residue in the peptide chain of the PG layer (13, 16, 35–37). Lpp-deficient *E. coli* strains possess significant OM defects, including release of periplasmic enzymes, increased levels of OM blebs, and increased sensitivity to certain compounds (16, 17). However, the phenotypes are mostly derived from laboratory-adapted strains of *E. coli* and, while a role for Lpp in serum resistance in a clinical *E. coli* isolate belonging to the ST131 strain group was recently demonstrated (21), the mechanism has remained undefined. Here, we provide unequivocal data supporting the idea of a critical role for Lpp in the uropathogenic *E. coli* CFT073 strain in preventing complement-mediated bacterial lysis *in vitro* and complement-mediated clearance *in vivo*. Lpp influences these functions, at least in part, by altering the cellular distribution of KpsD and consequently affecting the amount of K2 capsular polysaccharide, a known factor involved in CFT073 serum resistance (26). Group 2 capsules are typically produced by *E. coli* isolates from extraintestinal infections (extraintestinal pathogenic *E. coli* [ExPEC]), including those belonging to ST131 (38). The results of those studies also reinforce the idea of the importance of validating the role of bacterial proteins in clinical isolates with the full array of virulence determinants, so that *in vitro* phenotypes and interactions between cellular processes can be confirmed in animal models of bacterial infection.

Previous data demonstrated a role for cell surface polysaccharides (O-antigens and K antigens) and for Lpp and OMPs (OmpA and TraT) in resistance to serum killing (21, 26, 39), but the mechanisms by which they each act are unknown, as are any critical interactions between these components. Flow cytometry (see Fig. S2 in the supplemental material) and mass spectrometry proteomic analyses (see Table S5 in the supplemental material) clearly showed a substantial increase in deposition of complement components crucial for both classical and alternative complement pathways on the surface of CFT073 *lpp*, relative to WT CFT073 cells, confirming that Lpp inhibits bacterial lysis mediated by both complement pathways. The lower but detectable level of C3 deposited on WT CFT073 cells suggests that the alternative pathway “amplification loop,” which fixes C3b and allows factor B and factor D to enhance C3 deposition, may be inefficient against WT CFT073. Consistent with this, increased levels of properdin, a positive regulator of complement activation that binds to the alternative pathway C3 convertase and stabilizes the interaction of factor B and C3b (40, 41), were detected in CFT073 *lpp* membrane preparations relative to WT CFT073 after incubation with nHS but not HIHS (Table S5). Given that preexisting antibody-mediated immunity can significantly decrease the duration of UTIs (42), it is not surprising that UPEC isolates have evolved strategies to antagonize both alternative and classical complement activation pathways.

In addition to Lpp, several other proteins, including OmpA and Pal, have been demonstrated to interact noncovalently with the PG layer. Pal is a component of a larger multimeric Tol/Pal complex that includes periplasmic (TolB) and IM (TolA, TolQ, and TolR) proteins. TolB interacts with both TolA and Pal, thereby creating a structural link between the IM and OM (43). Interestingly, TolB expression levels were upregulated in CFT073 *lpp* cells relative to WT CFT073 (Table S3), perhaps suggesting that the cell was attempting to compensate for the loss of Lpp with alterative systems. While CFT073 *pal* was significantly attenuated *in vivo* (more so than CFT073 *lpp*; Fig. 1A and D), it remained resistant to serum *in vitro* (Fig. 1E). Survival of CFT073 *pal* cells could not be restored by infecting C3KO mice, indicating that attenuation of CFT073 *pal* involves effects that are independent of inhibition of complement-mediated bacterial lysis. Further investigation is needed to elucidate the exact mechanism(s).

KpsD is one of four conserved proteins required for transport of group 2 polysaccharides to the bacterial cell surface (44, 45). While early reports suggested KpsD is a periplasmic protein (33), later studies have indicated an OM location for (at least part of) the cellular pool of KpsD (45–47). In the working model, KpsD is an outer membrane translocon for polymer export (8, 31), and there are data supporting interactions between it and a cognate inner membrane protein (KpsE) (48), as part of a larger complex required for capsule biosynthesis and export (47). The reduction in KpsD observed in proteomics analyses was not due to a general downregulation of the system, because the amount of KpsE showed no apparent reduction in CFT073 *lpp* (Table S4); *kpsE* and *kpsD* (*kpsED*) are adjacent genes in the same transcriptional unit (Fig. 2B). This observation was surprising given that KpsD levels remained unchanged in total lysates or OM between WT and CFT073 *lpp* cells (Fig. 4D and E). This presumably reflects the existence of several forms of KpsD and selective loss in the different preparation methods. Interestingly, KpsD overexpression in CFT073 *lpp* cells led to increased amounts of KpsD in the SDS-insoluble fraction, although this was unable to rescue serum resistance in the absence of Lpp (Fig. 4G and H). Therefore, although Lpp is not essential for the association of KpsD with PG under conditions of expression at elevated (nonphysiological) levels, only Lpp-mediated interactions afford functional KpsD. Whether this reflects altered folding, oligomerization, or precise interaction of KpsD with PG in the absence of Lpp remains to be established. Previously, Arrecubieta et al. reported that localization of KpsD in the OM is dependent, in part, on the presence of Lpp and KpsE (46). Our data suggest that the amounts of KpsD in total cell lysates and the OM are comparable in WT CFT073 and CFT073 *lpp* but that Lpp instead maintains a previously unknown PG-associated form of KpsD that is correlated with high-level capsular polysaccharide expression. The differences in the two studies could reflect the isolates used. CFT073 is a clinical isolate, while the earlier studies (46) were performed using a hybrid *E. coli* K-12 strain carrying the capsule biosynthesis gene locus.

In summary, we have uncovered a novel mechanism by which Lpp contributes to resistance to complement-mediated bacterial lysis by influencing group 2 capsule assembly. The precise functional role of PG association in KpsD functionality is currently unknown, but it is conceivable that it is essential for productive assembly of a multiprotein export conduit that spans the cell envelope. KpsD (and KpsE) are conserved in all *E. coli* serotypes with group 2, and close homologs are found in *Campylobacter jejuni*, while related group 2 capsule assembly systems are present in other pathogens, including *Neisseria meningitidis* and *Haemophilus influenzae* (8). The role of Lpp in capsule assembly may therefore represent a shared mechanism. In this context, therapeutically targeting steps that inhibit Lpp synthesis and/or its transport to the OM (e.g., using MAC13243) (49) might, in combination with other antibacterials, lead to bacterial clearance in severely ill patients.

## MATERIALS AND METHODS

### Ethics statement.

All mice used in this study were housed and maintained at Genentech in accordance with American Association of Laboratory Animal Care guidelines. All experimental studies were conducted under protocols (no. 14-3397, 14-3643, and 16-0756) approved by the Institutional Animal Care and Use Committee of Genentech Lab Animal Research in an Association for Assessment and Accreditation of Laboratory Animal Care International (AAALAC)-accredited facility in accordance with the *Guide for the Care and Use of Laboratory Animals* and applicable laws and regulations.

### Bacterial strains and mutant construction.

The bacterial strains and plasmids used are listed in Table S6 in the supplemental material. *E. coli* strain CFT073 (ATCC 700928) (22) was purchased from ATCC. *E. coli* cells were grown in Luria-Bertani (LB) medium (0.5% yeast extract, 1% tryptone, 0.5% NaCl) at 37°C. Where indicated, kanamycin (Kan) was added to culture media at a 50 μg/ml final concentration. Gene disruption in CFT073 was performed as previously described (50). The primers used to generate the CFT073 mutants are listed in Table S7. Plasmids pKD46 for the λ Red recombinase (50), pKD4 for the integration construction (50), and pCP20 (51) for the FLP recombinase were used in this study.

10.1128/mBio.00603-17.10 TABLE S6  Bacterial strains and plasmids. Download TABLE S6, DOC file, 0.04 MB. Copyright © 2017 Diao et al. 2017 Diao et al. This content is distributed under the terms of the Creative Commons Attribution 4.0 International license.

10.1128/mBio.00603-17.11 TABLE S7  Primers used to generate CFT073 mutant strains. Download TABLE S7, DOC file, 0.03 MB. Copyright © 2017 Diao et al. 2017 Diao et al. This content is distributed under the terms of the Creative Commons Attribution 4.0 International license.

### Antibodies.

The anti-Pal and anti-YidC antibodies were generous gifts from Shaw Warren (Massachusetts General Hospital) and Eric Brown (McMaster University, Canada), respectively. The following remaining antibodies were obtained commercially unless stated otherwise: anti-GroEL (Enzo Life Sciences, Inc.); anti-K2 or K12 capsule antisera (Statens Serum Institut); anti-MBP (NEB); and anti-OmpA (Antibody Research Corporation). For initial experiments performed to detect Lpp expression, we used the anti-Lpp polyclonal antibody generated and given to us by Hajime Tokuda. Subsequently, we used our own anti-Lpp rabbit polyclonal antibody, the generation of which is described in Text S1 in the supplemental material. Expression of KpsD and production of the anti-KpsD antibody are also described in detail in Text S1.

10.1128/mBio.00603-17.1 TEXT S1  Supplemental text. Download TEXT S1, DOCX file, 0.1 MB. Copyright © 2017 Diao et al. 2017 Diao et al. This content is distributed under the terms of the Creative Commons Attribution 4.0 International license.

### *In vitro* growth and serum killing assays.

For *in vitro* growth curves, overnight cultures of WT CFT073, CFT073 *lpp*, and CFT073 *pal* were back diluted 1:100, grown to mid-exponential phase (optical density at 600 nm [OD_600_] = 0.6), and then diluted 1:100 to initiate growth curves. CFU levels were measured by plating on LB+Kan agar. Serum killing assays were carried out using normal human sera (nHS) pooled from five donors and were performed at a 100% serum concentration unless otherwise noted. For complement-depleted sera, the final concentration was 75%. As a control, nHS was heat inactivated (HIHS) at 56°C for 30 min. Sera deficient in specific complement components, as well as recombinant complement components, were purchased from Complement Technology and were used at the final concentration recommended by the manufacturer. Overnight cultures were back diluted 1:100 and grown to mid-exponential phase (OD_600_ = 0.6). Cells were collected by centrifugation at 3,000 × *g* for 10 min and resuspended in 100% nHS or HIHS and incubated for 30, 60, or 90 min at 37°C. At each time point, samples were diluted and plated on LB agar for CFU measurements.

### Mouse infection model.

Overnight bacterial cultures were back diluted 1:100 in M9 media and grown to an OD_600_ of 0.8 to 1 at 37°C. Cells were harvested, washed once with phosphate-buffered saline (PBS), and resuspended in PBS containing 10% glycerol. Cells were frozen in aliquots, and thawed aliquots were measured for CFU levels prior to mouse infections. Virulence of WT CFT073, CFT073 *lpp*, and CFT073 *pal* was measured using the neutropenic *E. coli* infection model (52). Briefly, 7-week-old A/J mice (Jackson Laboratory) were rendered neutropenic by peritoneal injection of 2 doses of cyclophosphamide (150 mg/kg of body weight on day −4 and 100 mg/kg on day −1). On day 0, mice were infected by intravenous injection through the tail vein of 5 × 10^5^ CFU mid-exponential-phase bacteria diluted in PBS. At 30 min and 24 h postinfection, bacterial burdens in the liver and spleen were determined by serial dilutions of tissue homogenates on LB plates.

For survival studies, eight-to-ten-week-old C57BL/6 or complement component 3-deficient mice (C3KO mice; Genentech) were rendered neutropenic and infected by intravenous injection through the tail vein of 3 × 10^7^ CFU mid-exponential-phase bacteria diluted in PBS. Mice were monitored for survival and body condition for 5 days.

### LPS analysis.

Bacterial cultures were grown to mid-exponential phase and then incubated with LB, nHS, HIHS, or complement component-depleted sera (with or without the respective recombinant complement component) as described above. Cells were collected by centrifugation at 3,000 × *g* for 10 min, resuspended in lysis buffer (2% [wt/vol] sodium dodecyl sulfate [SDS], 1 mM EDTA, 0.5 M Tris-HCl [pH 6.8]), and boiled for 10 min. Cell lysates were normalized based on OD_600_ and total protein levels and incubated with 10 μl of a 20 mg/ml stock of proteinase K (Sigma-Aldrich) at 60°C for 60 min. The extracts were mixed with sample buffer, boiled for 5 min, and separated by sodium dodecyl sulfate-polyacrylamide gel electrophoresis (SDS-PAGE). The gels were stained by the use of a Pro-Q emerald 300 LPS gel stain kit (Molecular Probes Inc.) to visualize LPS, as recommended by the manufacturer. The same samples were used to detect capsular polysaccharides by Western immunoblotting (details below).

### SDS-PAGE and Western immunoblotting.

Bacterial cell samples were resuspended in lysis buffer (4% SDS, 10 mM Tris-HCl [pH 7.5], 10 mM EDTA) and separated by SDS-PAGE (10% to 20% resolving gels), and Western blot analysis was performed using an iBlot 2 dry blotting system (Thermo Fisher Scientific). Polyvinylidene difluoride (PVDF) membranes were blocked in blocking buffer (PBS containing 5% bovine serum albumin [BSA] and 0.05% Tween 20) for 30 min and were then incubated overnight at 4°C with one of the following antibodies: rabbit anti-Lpp polyclonal antibody (1:1,000 final dilution); murine anti-Pal 6D7 antibody (0.5 μg/ml final concentration); rabbit anti-GroEL antibody (1:10,000 final dilution); anti-KpsD antibody (1:3,000 final dilution); rabbit anti-YidC antibody (1:200,000); rabbit anti-OmpA antibody (1:50,000); or rabbit anti-K2 and K12 capsule antisera (obtained from Statens SerumInstitut, Denmark; 1:500 final dilution). The membranes were washed with PBS–0.05% Tween-20 for 1 h and then incubated with secondary antibodies: donkey anti-rabbit antibody conjugated to horseradish peroxidase (Thermo Fisher Scientific) at a 1:10,000 final dilution; goat anti-rabbit antibody conjugated to alkaline phosphatase (Cedarlane Laboratories) at a 1:5,000 final dilution; and goat anti-mouse antibody conjugated to alkaline phosphatase (Jackson ImmunoResearch) at a 1:3,333 final dilution.

### Quantitation of K2 capsular polysaccharide by ELISA.

Capsular polysaccharide was quantitated using whole-cell ELISA as described previously (53). Briefly, mid-exponential-phase bacterial cultures were harvested by centrifugation at 2,500 × *g* for 5 min and resuspended to an OD_600_ of ~0.1 in PBS. Microtiter plates with 384 wells were coated with ~10^6^ intact bacteria at 25 μl/well and incubated overnight at 4°C. The following day, wells were incubated with 100 μl blocking buffer (PBS containing 5% BSA) followed by incubation with serial dilutions of rabbit anti-K2 or K12 antisera for 2 h at room temperature (RT). The plates were then washed and incubated with anti-rabbit IgG conjugated to alkaline phosphatase (Jackson ImmunoResearch Laboratories, Inc.) at a 1:2,000 final dilution for 1 h at RT. Wells were washed five times with PBS followed by addition of *p*-nitrophenyl phosphate (Sigma-Aldrich) for 30 min at RT. Reactions were stopped by addition of 50 μl of 3 N NaOH, and absorbance was read at 406 nm.

### Visualization and staining of WT CFT073, CFT073 *lpp*, and CFT073 *pal* by transmission electron microscopy.

Since bacterial capsular polysaccharides are significantly hydrated structures, visualization is technically challenging due to the dehydration artifacts involved during sample processing for electron microscopy (54). However, capsular polysaccharides can be stabilized for electron microscopy by cross-linking the surface glycans using antibodies (55, 56). Antibody stabilization and sample preparation were performed as previously described (27, 55) with some modifications. Bacteria were grown to mid-exponential phase and incubated with anti-K2 capsule antibody (1:1,000 dilution) for 30 min at RT, washed with PBS, and incubated in fixation buffer (0.1 M cacodylate buffer [pH 7.4], 5% glutaraldehyde, 0.15% ruthenium Red [Ted Pella, Inc.]), and stained. Cells were immobilized in 4% agarose (Sigma-Aldrich), washed 4 times at 10-min intervals in 0.1 M cacodylate buffer (pH 7.4) containing 0.05% ruthenium Red, and postfixed in 2% aqueous osmium tetroxide (Electron Microscopy Sciences) for 4 h, followed by wash steps using 0.1 M cacodylate buffer (pH 7.4). Samples were then dehydrated in sequential 30-min steps with increasing concentrations of acetone steps containing 0.05% ruthenium Red, followed by two 10-min exposures to propylene oxide, and were then embedded in Eponate 12 (Ted Pella, Inc.). Ultrathin (80-nm) sections were cut with an Ultracut microtome (Leica), stained with 0.5% uranyl acetate followed by 0.2% lead citrate for contrast, and examined using a Jeol JEM-1400 transmission electron microscope (TEM) at 80 kV. Digital images were captured with a Gatan Ultrascan 1000 charge-coupled-device (CCD) camera.

### Proteomic analyses of total bacterial membranes using liquid chromatography-tandem MS (LC/MS/MS).

Bacterial cultures (100 ml) were grown to mid-exponential phase, washed once with PBS, and resuspended in 6 ml PBSC (PBS containing cOmplete, mini, EDTA-free protease inhibitors). Cells were disrupted by two passages through a microfluidizer, and intact cells were removed from the lysate by centrifugation at 3,000 × *g* for 10 min. Membrane pellets were collected from the cell-free supernatant by centrifugation at 100,000 × *g* for 60 min, washed with PBS, and resuspended in 200 μl of PBSC. Samples were separated using SDS-PAGE, subjected to Coomassie blue staining, and cut into 10 pieces spanning the entire gel lane. Excised bands were washed in 50:50 acetonitrile:50 mM ammonium bicarbonate (Burdick and Jackson, Muskegon, MI). Gel slices were dehydrated with acetonitrile and then digested overnight at 37°C with 0.2 µg trypsin (Promega)–50 mM ammonium bicarbonate (pH 8). Peptides were extracted from gel bands using 50:50 acetonitrile:0.1% trifluoroacetic acid and evaporated to near-dryness before they were reconstituted in 10 µl of 2% acetonitrile–0.1% formic acid. Samples were injected via the use of an autosampler BEH µc_18_ column (Waters Corp) at a flow rate of 1 µl/min using a NanoAcquity ultraperformance liquid chromatography (UPLC) system (Waters Corp). A gradient from 98% solvent A (aqueous) to 80% solvent B (organic) was applied over 40 min. Samples were analyzed online via nanospray ionization into a hybrid linear trap quadrupole (LTQ)-Orbitrap mass spectrometer (Thermo Fisher Scientific). Data were collected in data-dependent mode, with the parent ion being analyzed in the Orbitrap and the top 8 most abundant ions being selected for fragmentation and analysis in the LTQ system. Tandem mass spectrometric data were analyzed using the Mascot search algorithm (Matrix Sciences, Boston, MA) against a UniProt.org database with specified *E. coli* and human taxonomies. Peptide hits are reported as “total peptides” and “unique peptides” based on spectral matches to putative sequences rather than a more quantitative method (area under the concentration-time curve [AUC]); hence, these data represent qualitative and not quantitative differences.

### Purification of peptidoglycan-associated proteins.

Purification of peptidoglycan-associated proteins was performed according to published methods (57, 58), with some modifications. Briefly, cells were harvested in mid-exponential phase, washed once, and resuspended in 6 ml of 10 mM Tris-HCl (pH 8.0). Cells were disrupted by two passages through an LV1 microfluidizer (Microfluidics). Intact cells were removed from the lysate by centrifugation at 3,000 × *g* for 10 min. Membrane pellets were collected and resuspended in 6 ml of 10 mM Tris-HCl (pH 8.0), containing 2% (wt/vol) SDS and cOmplete, mini, EDTA-free protease inhibitor cocktail (Sigma-Aldrich). The membrane preparations were subjected to another centrifugation at 100,000 × *g* for 30 min, and the pellet, containing peptidoglycan and associated proteins (including porins), was washed once with distilled water and resuspended in 300 μl of 50 mM Tris-HCl (pH 8.0) in a mixture containing 0.4 M NaCl, 5 mM EDTA, and protease inhibitor (referred to here as the SDS-insoluble fraction). The supernatant containing released cell envelope proteins was divided into aliquots and frozen (referred to here as the SDS-soluble fraction). Both fractions were subjected to Western immunoblotting as described above.

### IM and OM isolation and sucrose gradient centrifugation.

Cultures (100 ml) were grown at 37°C with shaking until they reached mid-log phase (OD_600_ of ~0.6). Cells were harvested by centrifugation (5,000 × *g*, 10 min, 4°C), and cell pellets were resuspended in 20 ml 5 mM EDTA (pH 8.0) and lysed by passage through a French press. Unbroken cells were removed by centrifugation for 10 min at 10,000 × *g* at 4°C before membrane pellets were collected at 100,000 × *g* at 4°C. Membranes were resuspended, and fractionation was performed as previously described (59). Further details are provided in Text S1. IM and IM-free OM fractions from the sucrose gradients were analyzed by SDS PAGE and Western immunoblotting.

### Statistical analyses.

All statistical analyses were performed using GraphPad Prism software (GraphPad). All graphs represent means ± standard errors of the means (SEM). Unless stated otherwise, *P* values for all data were determined using regular unpaired *t* tests (*, *P* < 0.05; **, *P* < 0.01; ***, *P* < 0.001). The data were tested for being parametric, and statistical analyses were performed on log-transformed data. *P* values for mouse lethality studies were determined using log rank (Mantel-Cox) tests and were adjusted for multiple testing using Bonferroni correction.
