# Supplementary material for: Peptidoglycan Association of Murein Lipoprotein Is Required for KpsD-Dependent Group 2 Capsular Polysaccharide Expression and Serum Resistance in a Uropathogenic Escherichia coli Isolate
Source: mBio. 2017 May 23;8(3):e00603-17. doi: 10.1128/mBio.00603-17 (PMC5442458; doi:10.1128/mBio.00603-17)
Supplement: TABLE S1 [file mbo003173319st1.doc]

**Table S1:** Minimal inhibitory concentrations (MIC) of known antibacterials and other compounds against WT CFT073, CFT073*lpp* and CFT073*pal*

| Antibiotics/Compounds | MIC (μM unless stated otherwise) | | |
| --- | --- | --- | --- |
|  | WT CFT073 | CFT073*lpp* | CFT073*pal* |
| Tetracycline | 9.4 + 4.4 | 9.4 + 4.4 | 9.4 + 4.4 |
| Chloramphenicol | 25 | 18.8 + 8.8 | 12.5 |
| Perfloxacin | 0.8 | 0.8 | 0.8 |
| Polymyxin B | 0.2 | 0.2 | 0.2 |
| Colistin | 0.1 | 0.1 | 0.1 |
| Levofloxacin | 0.4 | 0.4 | 0.8 |
| Norfloxacin | 0.2 | 0.2 | 0.2 |
| Doripenem | 0.2 | 0.2 | 0.2 |
| Gentamicin (μg/ml) | 0.7 + 0.3 | 0.7 + 0.3 | 0.5 |
| Carbenicillin (μg/ml) | 0.2 | 0.2 | 0.2 |
| Hygromycin B (μg/ml) | 15.6 | 15.6 | 15.6 |
| Bile salts (μg/ml) | 18.8 + 8.8 | 12.5 | 12.5 |
| SDS (%) | 1.3 | 0.3 | 0.6 |
| Spectinomycin | 100 | 100 | 100 |
| Pseudomonic acid | 100 | 100 | 100 |
| Linezolid | >100 | >100 | >100 |
| Bacitracin | >100 | >100 | >100 |
| Sulfaguanidine | >100 | >100 | >100 |
| fosfomycin | >100 | >100 | >100 |
| Erythromycin | >100 | 100 | 25 |
| Oxacillin | >100 | >100 | >100 |
| Clindamycin | >100 | >100 | >100 |
| Vancomycin | 100 | >100 | 100 |
| Streptomycin | >100 | >100 | >100 |
| Actinomycin D | >100 | >100 | 100 |
| Doxurubicin | >100 | >100 | >100 |

Data are representative (Mean + SEM) for two independent replicates
