# Supplementary material for: Peptidoglycan Association of Murein Lipoprotein Is Required for KpsD-Dependent Group 2 Capsular Polysaccharide Expression and Serum Resistance in a Uropathogenic Escherichia coli Isolate
Source: mBio. 2017 May 23;8(3):e00603-17. doi: 10.1128/mBio.00603-17 (PMC5442458; doi:10.1128/mBio.00603-17)
Supplement: TABLE S2 [file mbo003173319st2.docx]

**Table S2**: Levels of membrane-associated bacterial proteins decreased in CFT073*lpp* versus WT CFT073 identified through LC-MS

| Uniprot | Description | Gene | CL ^a^ | Total (Unique) Peptides | | | | | |
| --- | --- | --- | --- | --- | --- | --- | --- | --- | --- |
|  |  |  |  | CFT073  (LB) | *lpp*  (LB) | CFT073  (nHS) | *lpp*  (nHS) | CFT073  (HIHS) | *lpp*  (HIHS) |
| LPP_ECOL6 | Lpp (murein-lipoprotein) | lpp | OM | 257 (15) | 3 (4) | 276 (15) | 2 (2) | 263 (17) | 3 (3) |
| Q8FDQ1_ECOL6 | KpsD | kpsD | OM/P | 47 (31) | 13 (12) | 52 (28) | 26 (20) | 42 (32) | 30 (21) |
| Q8FKJ3_ECOLI | Uncharacterized protein (UpaB) | c0426 | OM | 27 (19) | 7 (6) | 24 (18) | 5 (5) | 20 (16) | 6 (5) |
| Q8CW66_ECOL6 | UpaD | c1273 | - | 22 (20) | 6 (5) | 18 (14) | 5 (5) | 19 (18) | 2 (2) |
| Q8CVG1_ECOL6 | Outer membrane usher protein FimD | fimD | OM | 21 (19) | 9 (8) | 23 (21) | 8 (8) | 21 (18) | 9 (9) |
| Q8FHH8_ECOL6 | Formate dehydrogenase, major subunit | fdnG | p-IM | 20 (20) | 4 (4) | 19 (18) | 4 (4) | 19 (19) | 1 (1) |
| Q8FCT1_ECOL6 | 4-alpha-glucanotransferase | malQ | p-IM | 20 (20) | 11 (11) | 23 (22) | 10 (7) | 18 (18) | 13 (13) |
| Q8FHT4_ECOL6 | Enoyl-[acyl-carrier-protein] reductase | FabI | p-IM | 18 (16) | 9 (9) | 19 (17) | 7 (6) | 15 (14) | 8 (8) |
| Q8CVQ8_ECOL6 | Hydrogenase-2 large chain | hybC | p-IM | 18 (15) | 8 (8) | 14 (12) | 9 (9) | 17 (17) | 14 (11) |
| Q8FG27_ECOL6 | Phosphomannomutase | cpsG | - | 16 (14) | 3 (3) | 18 (15) | 7 (7) | 21 (18) | 10 (9) |
| SYS_ECOL6 | Seryl-tRNA synthetase SerRS | serS | IM | 9 (9) | 4 (4) | 8 (8) | 1 (1) | 7 (7) | 2 (2) |
| SYH_ECOL6 | Histidyl-tRNA synthetase HisRS | hisS | p-IM | 9 (7) | 2 (2) | 11 (9) | 4 (4) | 9 (5) | 7 (6) |
| Q8CVT9_ECOL6 | Uncharacterized protein | yfeY | OM | 6 (6) | 3 (3) | 7 (7) | 4 (4) | 7 (7) | 4 (4) |
| ACKA_ECOL6 | Acetate kinase (Acetokinase) | ackA | p-IM | 5 (4) | 2 (2) | 12 (10) | 2 (2) | 8 (5) | 3 (3) |

CL ^a^ = cellular localization; OM=outer membrane; IM=inner membrane; p-IM=peripherally localized to the IM; P=periplasm; “-“=no localization annotated in *STEP*db (<http://www.stepdb.eu/step2/>)
