# Supplementary material for: Peptidoglycan Association of Murein Lipoprotein Is Required for KpsD-Dependent Group 2 Capsular Polysaccharide Expression and Serum Resistance in a Uropathogenic Escherichia coli Isolate
Source: mBio. 2017 May 23;8(3):e00603-17. doi: 10.1128/mBio.00603-17 (PMC5442458; doi:10.1128/mBio.00603-17)
Supplement: TABLE S3 [file mbo003173319st3.docx]

**Table S3**: Levels of membrane-associated bacterial proteins increased in CFT073*lpp* versus WT CFT073 identified through LC-MS

| Uniprot | Description | Gene | CL ^a^ | Total (Unique) Peptides | | | | | | |
| --- | --- | --- | --- | --- | --- | --- | --- | --- | --- | --- |
|  |  |  |  | CFT073  (LB) | | *lpp*  (LB) | CFT073  (nHS) | *lpp*  (nHS) | CFT073  (HIHS) | *lpp*  (HIHS) |
| MUKB_ECOLI | Chromosome partition protein MukB | mukB | p-IM | | 32 (30) | 64 (47) | 20 (20) | 57 (39) | 28 (25) | 46 (39) |
| Q8FCS4_ECOL6 | Glycerol-3-phosphate dehydrogenase | glpD | p-IM | | 21 (19) | 56 (35) | 23 (20) | 56 (35) | 21 (18) | 52 (37) |
| MREB_ECOL6 | Rod shape-determining protein mreB | mreB | p-IM | | 10 (9) | 34 (15) | 8 (8) | 27 (16) | 9 (9) | 21 (15) |
| Q8FDF8_ECOLI | Aerotaxis receptor | aer | IM | | 8 (6) | 20 (15) | 6 (5) | 22 (19) | 7 (6) | 17 (14) |
| LLDD_ECOL6 | L-lactate dehydrogenase | lldD | p-IM | | 6 (6) | 24 (17) | 8 (8) | 29 (19) | 8 (8) | 27 (20) |
| SRP54_ECOLI | Signal recognition particle protein | ffh | p-IM | | 6 (6) | 24 (21) | 9 (7) | 22 (20) | 8 (8) | 24 (21) |
| Q8FAB6_ECOL6 | Uncharacterized protein | c5378 | p-IM | | 6 (6) | 17 (16) | 2 (2) | 10 (10) | 4 (4) | 11 (10) |
| AAS_ECOLI | Bifunctional protein Aas | aas | IM | | 5 (5) | 11 (11) | 10 (10) | 16 (13) | 8 (7) | 19 (15) |
| Q8FFW1_ECOLI | D-lactate dehydrogenase | dld | p-IM | | 5 (4) | 13 (11) | 7 (7) | 25 (23) | 10 (8) | 25 (22) |
| Q8FFN6_ECOL6 | Glycerol-3-phosphate transporter | glpT | IM | | 5 (3) | 11 (8) | 11 (11) | 17 (12) | 5 (5) | 11 (11) |
| Q8FCP0_ECOLI | Signal recognition particle receptor FtsY | ftsY | p-IM | | 4 (4) | 10 (10) | 1 (1) | 9 (8) | 3 (3) | 8 (8) |
| OPGH_ECOL6 | Glucans biosynthesis glucosyltransferase H | mdoH | IM | | 4 (4) | 19 (15) | 3 (3) | 31 (25) | 5 (3) | 22 (16) |
| TOLB_ECOL6 | Protein tolB | tolB | P | | 3 (2) | 11 (9) | 13 (9) | 23 (17) | 7 (6) | 25 (17) |
| Q8FH47_ECOLI | Uncharacterized protein | ydiJ | p-IM | | 3 (3) | 28 (19) | 5 (5) | 31 (24) | 3 (3) | 27 (18) |
| Q8CVT2_ECOLI | Hypothetical lipoprotein yfhM | yfhM | IM | | 2 (2) | 11 (10) | 1 (1) | 13 (11) | 3 (3) | 1 (1) |
| DADA_ECOL6 | D-amino acid dehydrogenase small subunit | dadA | p-IM | | 2 (2) | 10 (9) | 2 (2) | 8 (7) | 3 (2) | 5 (5) |
| RSXC_ECOLI | Electron transport complex subunit RsxC | rsxC | - | | 1 (1) | 15 (13) | 3 (3) | 14 (11) | 3 (3) | 12 (9) |
| LCFA_ECOL6 | Long-chain-fatty-acid--CoA ligase | fadD | IM | | 1 (1) | 10 (7) | 2 (2) | 7 (5) | 1 (1) | 8 (5) |
| SURA_ECOLI | Chaperone SurA | surA | P | | 0 (0) | 7 (7) | 2 (2) | 17 (16) | 5 (5) | 15 (14) |
| OPGB_ECOLI | Phosphoglycerol transferase I | mdoB | IM | | 0 (0) | 6 (6) | 0 (0) | 6 (6) | 0 (0) | 2 (2) |

CL ^a^ = cellular localization; OM=outer membrane; IM=inner membrane; p-IM=peripherally localized to the IM; P=periplasm; “-“=no localization annotated in *STEP*db (<http://www.stepdb.eu/step2/>)
