# Supplementary material for: Peptidoglycan Association of Murein Lipoprotein Is Required for KpsD-Dependent Group 2 Capsular Polysaccharide Expression and Serum Resistance in a Uropathogenic Escherichia coli Isolate
Source: mBio. 2017 May 23;8(3):e00603-17. doi: 10.1128/mBio.00603-17 (PMC5442458; doi:10.1128/mBio.00603-17)
Supplement: TABLE S4 [file mbo003173319st4.docx]

**Table S4:** Membrane-associated bacterial proteins expressed at similar levels in CFT073*lpp* versus WT CFT073 identified through LC/MS/MS

| Uniprot | Description | Gene | CL ^a^ | Total (Unique) Peptides | | | | | |
| --- | --- | --- | --- | --- | --- | --- | --- | --- | --- |
|  |  |  |  | CFT073  (LB) | *lpp*  (LB) | CFT073  (nHS) | *lpp*  (nHS) | CFT073  (HIHS) | *lpp*  (HIHS) |
| 6PGD_ECOLI | 6-phosphogluconate dehydrogenase, decarboxylating | gnd | p-IM | 1 (1) | 1 (1) | 6 (6) | 0 (0) | 3 (3) | 0 (0) |
| AAS_ECOLI | Bifunctional protein Aas | aas | IM | 5 (5) | 11 (11) | 10 (10) | 16 (13) | 8 (7) | 19 (15) |
| ACCA_ECOLI | Acetyl-coenzyme A carboxylase carboxyl transferase subunit alpha | accA | p-IM | 7 (5) | 6 (4) | 6 (5) | 3 (3) | 3 (2) | 6 (4) |
| ACCC_ECOLI | Biotin carboxylase | accC | p-IM | 20 (18) | 26 (22) | 19 (18) | 16 (15) | 19 (18) | 21 (19) |
| ACNB_ECOLI | Aconitate hydratase 2 | acnB | p-IM | 21 (16) | 23 (16) | 14 (12) | 13 (10) | 22 (17) | 24 (16) |
| ACRA_ECOLI | Acriflavine resistance protein A | acrA | IM | 24 (17) | 33 (19) | 29 (19) | 30 (19) | 25 (18) | 31 (18) |
| ACRB_ECOLI | Acriflavine resistance protein B | acrB | IM | 41 (33) | 55 (26) | 59 (34) | 67 (43) | 46 (36) | 40 (30) |
| ADHE_ECOLI | Aldehyde-alcohol dehydrogenase | adhE | p-IM | 112 (49) | 68 (35) | 127 (50) | 88 (41) | 119 (50) | 83 (39) |
| AHPC_ECOLI | Alkyl hydroperoxide reductase subunit C | ahpC | p-IM | 5 (5) | 1 (1) | 3 (3) | 4 (3) | 4 (4) | 7 (6) |
| AK2H_ECOLI | AKII-HDII protein | metL | p-IM | 1 (1) | 1 (1) | 2 (2) | 2 (2) | 4 (4) | 1 (1) |
| AMPA_ECOLI | Cytosol aminopeptidase | pepA | p-IM | 5 (5) | 11 (10) | 7 (7) | 9 (8) | 11 (10) | 11 (10) |
| AMPE_ECOLI | AmpE protein | ampE | IM | 1 (1) | 4 (4) | 2 (2) | 7 (7) | 4 (4) | 2 (2) |
| ARCA_ECOLI | Aerobic respiration control protein ArcA | arcA | p-IM | 13 (12) | 7 (5) | 8 (6) | 1 (1) | 6 (5) | 3 (3) |
| ARCB_ECOLI | Aerobic respiration control sensor protein arcB | arcB | IM | 5 (5) | 10 (9) | 4 (4) | 11 (11) | 5 (5) | 7 (7) |
| ASPA_ECOLI | Aspartate ammonia-lyase | aspA | p-IM | 14 (12) | 11 (8) | 19 (15) | 9 (8) | 14 (13) | 12 (11) |
| ATKA_ECOLI | Potassium-transporting ATPase A chain | kdpA | IM | 0 (0) | 0 (0) | 1 (1) | 1 (1) | 1 (1) | 4 (4) |
| ATKB_ECOLI | Potassium-transporting ATPase B chain | kdpB | IM | 45 (25) | 35 (23) | 47 (30) | 45 (26) | 41 (28) | 40 (24) |
| ATP6_ECOLI | ATP synthase subunit a | atpB | IM | 1 (1) | 3 (2) | 0 (0) | 1 (1) | 1 (1) | 3 (2) |
| ATPA_ECOLI | ATP synthase subunit alpha | atpA | p-IM | 72 (35) | 110 (36) | 65 (34) | 110 (34) | 71 (34) | 102 (35) |
| ATPB_ECOLI | ATP synthase subunit beta | atpD | p-IM | 49 (36) | 50 (34) | 45 (33) | 44 (32) | 50 (33) | 48 (34) |
| ATPD_ECOLI | ATP synthase subunit delta | atpH | p-IM | 4 (3) | 4 (3) | 0 (0) | 5 (4) | 0 (0) | 3 (2) |
| ATPE_ECOLI | ATP synthase epsilon chain | atpC | p-IM | 1 (1) | 0 (0) | 1 (1) | 1 (1) | 1 (1) | 0 (0) |
| ATPF_ECOLI | ATP synthase subunit b | atpF | IM | 31 (16) | 36 (16) | 30 (17) | 38 (18) | 33 (19) | 30 (20) |
| ATPG_ECOLI | ATP synthase gamma chain | atpG | p-IM | 16 (13) | 19 (15) | 21 (16) | 27 (18) | 18 (15) | 25 (17) |
| ATZN_ECOLI | Lead, cadmium, zinc and mercury transporting ATPase | zntA | IM | 7 (6) | 16 (13) | 9 (7) | 14 (13) | 7 (6) | 14 (10) |
| BAMA_ECOLI | Outer membrane protein assembly factor BamA | bamA | OM | 155 (53) | 166 (52) | 172 (53) | 160 (52) | 172 (52) | 145 (49) |
| BAMB_ECOLI | Outer membrane protein assembly factor BamB | yfgL | OM | 41 (22) | 37 (18) | 46 (21) | 42 (19) | 47 (22) | 42 (19) |
| BAMC_ECOLI | Outer membrane protein assembly factor BamC | nlpB | OM | 39 (18) | 43 (18) | 46 (22) | 38 (19) | 41 (19) | 48 (20) |
| BAMD_ECOLI | Outer membrane protein assembly factor BamD | bamD | OM | 32 (17) | 29 (18) | 28 (19) | 33 (20) | 42 (17) | 34 (20) |
| BEPA_ECOLI | Uncharacterized protein | yfgC | P | 1 (1) | 2 (2) | 0 (0) | 1 (1) | 0 (0) | 2 (2) |
| BTUB_ECOLI | Vitamin B12 transporter BtuB | btuB | OM | 65 (34) | 52 (27) | 65 (34) | 60 (35) | 68 (36) | 48 (30) |
| CADB_ECOLI | Probable cadaverine/lysine antiporter | cadB | IM | 3 (3) | 4 (4) | 3 (3) | 3 (3) | 4 (4) | 2 (2) |
| CAPP_ECOLI | Phosphoenolpyruvate carboxylase | ppc | p-IM | 1 (1) | 4 (3) | 4 (4) | 13 (13) | 6 (6) | 8 (8) |
| CARA_ECOLI | Carbamoyl-phosphate synthase small chain | carA | p-IM | 1 (1) | 1 (1) | 1 (1) | 0 (0) | 0 (0) | 1 (1) |
| CARB_ECOLI | Carbamoyl-phosphate synthase large chain | carB | p-IM | 36 (34) | 26 (25) | 25 (25) | 22 (21) | 27 (27) | 27 (26) |
| CCMC_ECOLI | Heme exporter protein C | ccmC | IM | 1 (1) | 1 (1) | 1 (1) | 2 (2) | 0 (0) | 1 (1) |
| CCMF_ECOLI | Cytochrome c-type biogenesis protein ccmF | ccmF | IM | 2 (2) | 3 (3) | 3 (2) | 4 (3) | 2 (2) | 2 (2) |
| CDH_ECOLI | CDP-diacylglycerol pyrophosphatase | cdh | P | 5 (5) | 8 (7) | 8 (8) | 9 (8) | 5 (4) | 2 (2) |
| CH60_ECOLI | 60 kDa chaperonin | groL | p-IM | 48 (27) | 43 (23) | 38 (23) | 18 (13) | 27 (19) | 23 (18) |
| CHEA_ECOLI | Histidine kinase | cheA | p-IM | 10 (10) | 7 (7) | 10 (8) | 6 (6) | 6 (5) | 8 (8) |
| CHEZ_ECOLI | Protein phosphatase CheZ | cheZ | p-IM | 2 (2) | 4 (3) | 1 (1) | 1 (1) | 2 (2) | 4 (3) |
| CIRA_ECOLI | Colicin I receptor | cirA | OM | 8 (7) | 10 (8) | 7 (6) | 6 (6) | 5 (4) | 4 (3) |
| CISY_ECOLI | Citrate synthase | gltA | p-IM | 1 (1) | 7 (7) | 5 (5) | 5 (5) | 4 (4) | 6 (6) |
| CLPB_ECOLI | Chaperone protein ClpB | clpB | p-IM | 19 (19) | 12 (12) | 11 (10) | 12 (12) | 13 (13) | 23 (22) |
| COBS_ECOLI | Adenosylcobinamide-GDP ribazoletransferase | cobS | IM | 0 (0) | 2 (2) | 1 (1) | 3 (3) | 1 (1) | 2 (2) |
| CODB_ECOLI | Cytosine permease | codB | IM | 4 (4) | 4 (4) | 4 (4) | 5 (5) | 4 (4) | 4 (4) |
| CORA_ECOLI | Magnesium transport protein CorA | corA | IM | 4 (4) | 3 (3) | 5 (4) | 4 (4) | 5 (5) | 4 (3) |
| CPXA_ECOLI | Sensor protein CpxA | cpxA | IM | 2 (2) | 5 (5) | 4 (4) | 10 (10) | 4 (4) | 8 (8) |
| CRP_ECOLI | cAMP-activated global transcriptional regulator CRP | crp | p-IM | 8 (8) | 12 (12) | 6 (6) | 8 (8) | 6 (6) | 11 (11) |
| CSTA_ECOLI | Carbon starvation protein A | cstA | IM | 0 (0) | 2 (2) | 0 (0) | 6 (4) | 2 (2) | 4 (4) |
| CYDA_ECOLI | Cytochrome bd-I ubiquinol oxidase subunit 1 | cydA | IM | 55 (12) | 61 (12) | 59 (12) | 79 (12) | 66 (12) | 58 (13) |
| CYDB_ECOLI | Cytochrome bd-I ubiquinol oxidase subunit 2 | cydB | IM | 8 (4) | 8 (4) | 12 (4) | 12 (4) | 6 (4) | 8 (3) |
| CYDC_ECOLI | Transport ATP-binding protein cydC | cydC | IM | 5 (5) | 5 (5) | 7 (7) | 10 (10) | 7 (7) | 11 (10) |
| CYDD_ECOLI | Transport ATP-binding protein cydD | cydD | IM | 8 (8) | 14 (12) | 9 (9) | 13 (13) | 9 (9) | 12 (11) |
| CYOA_ECOLI | Cytochrome bo(3) ubiquinol oxidase subunit 2 | cyoA | IM, IM | 15 (12) | 21 (14) | 16 (13) | 31 (15) | 16 (14) | 25 (15) |
| CYOB_ECOLI | Cytochrome bo(3) ubiquinol oxidase subunit 1 | cyoB | IM | 11 (7) | 20 (12) | 12 (9) | 19 (14) | 15 (11) | 17 (10) |
| CYSA_ECOLI | Sulfate/thiosulfate import ATP-binding protein CysA | cysA | p-IM | 2 (2) | 3 (3) | 2 (2) | 3 (3) | 2 (2) | 4 (4) |
| CYSZ_ECOLI | Sulfate transporter CysZ | cysZ | IM | 2 (2) | 6 (6) | 3 (3) | 5 (5) | 2 (2) | 6 (6) |
| DACA_ECOLI | D-alanyl-D-alanine carboxypeptidase DacA | dacA | p-IM | 23 (20) | 24 (19) | 23 (17) | 40 (24) | 19 (15) | 38 (24) |
| DACC_ECOLI | Penicillin-binding protein 6 | dacC | p-IM | 12 (11) | 17 (15) | 14 (13) | 25 (21) | 16 (16) | 19 (15) |
| DAMX_ECOLI | DamX protein | damX | IM | 19 (13) | 21 (14) | 17 (12) | 23 (15) | 14 (11) | 21 (14) |
| DBHA_ECOLI | DNA-binding protein HU-alpha | hupA | p-IM | 4 (3) | 1 (1) | 2 (1) | 0 (0) | 0 (0) | 0 (0) |
| DCTA_ECOLI | Aerobic C4-dicarboxylate transport protein | dctA | IM | 4 (4) | 6 (4) | 5 (4) | 6 (4) | 4 (4) | 7 (4) |
| DCUA_ECOLI | Anaerobic C4-dicarboxylate transporter DcuA | dcuA | IM | 6 (4) | 7 (4) | 11 (8) | 8 (4) | 10 (4) | 6 (4) |
| DCUB_ECOLI | Anaerobic C4-dicarboxylate transporter DcuB | dcuB | IM | 3 (3) | 2 (2) | 3 (3) | 2 (2) | 3 (3) | 2 (2) |
| DCUC_ECOLI | Anaerobic C4-dicarboxylate transporter DcuC | dcuC | IM | 3 (3) | 0 (0) | 2 (2) | 1 (1) | 2 (2) | 0 (0) |
| DCUS_ECOLI | Sensor histidine kinase DcuS | dcuS | IM | 1 (1) | 3 (3) | 1 (1) | 3 (3) | 0 (0) | 4 (4) |
| DEAD_ECOLI | ATP-dependent RNA helicase DeaD | deaD | p-IM | 16 (15) | 14 (12) | 13 (13) | 19 (17) | 19 (16) | 17 (14) |
| DEDD_ECOLI | DedD protein | dedD | IM | 6 (6) | 8 (7) | 10 (7) | 10 (8) | 7 (5) | 9 (7) |
| DER_ECOLI | GTPase Der | der | p-IM | 15 (14) | 10 (9) | 13 (12) | 6 (6) | 6 (6) | 10 (10) |
| DHG_ECOLI | Glucose dehydrogenase | gcd | IM | 14 (14) | 16 (14) | 15 (14) | 21 (19) | 16 (14) | 14 (13) |
| DHNA_ECOLI | NADH dehydrogenase | ndh | p-IM | 21 (18) | 36 (20) | 34 (24) | 63 (30) | 34 (22) | 31 (21) |
| DHSA_ECOLI | Succinate dehydrogenase flavoprotein subunit | sdhA | p-IM | 22 (19) | 29 (22) | 30 (22) | 28 (21) | 24 (19) | 29 (23) |
| DHSB_ECOLI | Succinate dehydrogenase iron-sulfur subunit | sdhB | p-IM | 9 (9) | 17 (14) | 10 (7) | 15 (10) | 9 (9) | 14 (11) |
| DHSC_ECOLI | Succinate dehydrogenase cytochrome b-556 subunit | sdhC | IM | 8 (4) | 13 (7) | 11 (4) | 13 (6) | 11 (3) | 9 (4) |
| DHSD_ECOLI | Succinate dehydrogenase hydrophobic membrane anchor subunit | sdhD | IM | 9 (4) | 6 (4) | 7 (4) | 11 (4) | 8 (4) | 8 (4) |
| DJLA_ECOLI | DnaJ-like protein DjlA | djlA | IM | 5 (5) | 6 (6) | 5 (5) | 6 (6) | 5 (5) | 3 (3) |
| DLDH_ECOLI | Dihydrolipoyl dehydrogenase | lpdA | p-IM | 42 (25) | 46 (27) | 31 (22) | 45 (24) | 32 (22) | 35 (24) |
| DNAJ_ECOLI | Chaperone protein DnaJ | dnaJ | p-IM | 9 (9) | 15 (12) | 9 (9) | 13 (12) | 13 (12) | 12 (10) |
| DNAK_ECOLI | Chaperone protein DnaK | dnaK | p-IM | 6 (6) | 12 (10) | 18 (17) | 19 (15) | 21 (16) | 26 (21) |
| DSBB_ECOLI | Disulfide bond formation protein B | dsbB | IM | 1 (1) | 2 (2) | 2 (2) | 2 (2) | 3 (3) | 0 (0) |
| DSBD_ECOLI | Thiol:disulfide interchange protein DsbD | dsbD | IM | 0 (0) | 1 (1) | 2 (1) | 4 (2) | 0 (0) | 5 (3) |
| DSDX_ECOLI | DsdX permease | dsdX | IM | 9 (8) | 10 (8) | 9 (7) | 7 (6) | 10 (9) | 7 (5) |
| EFG_ECOLI | Elongation factor G | fusA | p-IM | 32 (26) | 27 (20) | 34 (26) | 31 (26) | 33 (28) | 32 (24) |
| EFTS_ECOLI | Elongation factor Ts | tsf | p-IM | 4 (4) | 0 (0) | 1 (1) | 3 (3) | 4 (4) | 12 (8) |
| EFTU1_ECOLI | Elongation factor Tu | tufA | p-IM | 103 (34) | 89 (27) | 75 (32) | 60 (25) | 73 (31) | 107 (28) |
| EMRA_ECOLI | Multidrug resistance protein A | emrA | IM | 11 (11) | 11 (11) | 14 (12) | 17 (16) | 13 (13) | 13 (12) |
| EMTA_ECOLI | Endo-type membrane-bound lytic murein transglycosylase A | emtA | OM | 3 (3) | 4 (4) | 5 (5) | 2 (2) | 4 (4) | 4 (4) |
| ENVZ_ECOLI | Histidine kinase | envZ | IM | 0 (0) | 2 (2) | 0 (0) | 5 (5) | 1 (1) | 0 (0) |
| EXBB_ECOLI | Biopolymer transport exbB protein | exbB | IM | 5 (4) | 5 (4) | 5 (4) | 6 (5) | 5 (4) | 5 (4) |
| FABB_ECOLI | 3-oxoacyl-[acyl-carrier-protein] synthase 1 | fabB | p-IM | 5 (5) | 4 (4) | 5 (5) | 5 (5) | 6 (6) | 3 (3) |
| FABZ_ECOLI | 3-hydroxyacyl-[acyl-carrier-protein] dehydratase FabZ | fabZ | p-IM | 6 (6) | 8 (5) | 9 (8) | 8 (7) | 10 (7) | 6 (4) |
| FADJ_ECOLI | Fatty acid oxidation complex subunit alpha | fadJ | p-IM | 1 (1) | 0 (0) | 0 (0) | 0 (0) | 0 (0) | 1 (1) |
| FADL_ECOLI | Long-chain fatty acid transport protein | fadL | OM | 53 (22) | 48 (18) | 44 (17) | 45 (17) | 60 (21) | 41 (16) |
| FDOG_ECOLI | Formate dehydrogenase-O, major subunit | fdoG | P | 25 (20) | 30 (24) | 27 (24) | 47 (38) | 32 (26) | 39 (28) |
| FDOH_ECOLI | Formate dehydrogenase-O iron-sulfur subunit | fdoH | p-IM | 0 (0) | 2 (2) | 2 (2) | 9 (8) | 2 (2) | 1 (1) |
| FDOI_ECOLI | Formate dehydrogenase, cytochrome b556(fdo) subunit | fdoI | IM | 5 (4) | 7 (5) | 3 (3) | 6 (5) | 3 (3) | 5 (3) |
| FEPA_ECOLI | Ferrienterobactin receptor | fepA | OM | 12 (12) | 11 (10) | 11 (11) | 9 (7) | 13 (12) | 6 (5) |
| FHUA_ECOLI | Ferrichrome-iron receptor | fhuA | OM | 84 (42) | 68 (28) | 80 (44) | 78 (39) | 83 (44) | 50 (31) |
| FIMA1_ECOLI | Type-1 fimbrial protein, A chain | fimA | OM | 3 (3) | 1 (1) | 5 (2) | 5 (1) | 3 (2) | 3 (1) |
| FIMC_ECOLI | Chaperone protein FimC | fimC | P | 3 (3) | 3 (3) | 6 (4) | 3 (3) | 5 (3) | 2 (2) |
| FIS_ECOLI | DNA-binding protein Fis | fis | p-IM | 5 (4) | 6 (4) | 5 (4) | 5 (4) | 6 (4) | 4 (4) |
| FIU_ECOLI | Catecholate siderophore receptor Fiu | fiu | OM | 14 (13) | 16 (15) | 14 (14) | 18 (17) | 15 (14) | 12 (11) |
| FKBA_ECOLI | FKBP-type peptidyl-prolyl cis-trans isomerase FkpA | fkpA | P | 0 (0) | 0 (0) | 3 (3) | 7 (5) | 2 (1) | 1 (1) |
| FLGE_ECOLI | Flagellar hook protein flgE | flgE | OM | 18 (11) | 22 (12) | 20 (12) | 23 (11) | 20 (11) | 24 (11) |
| FLGG_ECOLI | Flagellar basal-body rod protein FlgG | flgG | P | 6 (6) | 6 (5) | 8 (8) | 5 (5) | 5 (5) | 7 (7) |
| FLGH_ECOLI | Flagellar L-ring protein | flgH | OM | 20 (8) | 14 (7) | 13 (8) | 12 (6) | 21 (8) | 13 (5) |
| FLGI_ECOLI | Flagellar P-ring protein | flgI | P | 12 (11) | 11 (10) | 13 (12) | 12 (11) | 10 (9) | 13 (13) |
| FLGK_ECOLI | Flagellar hook-associated protein 1 | flgK | OM | 5 (4) | 11 (10) | 8 (6) | 13 (13) | 6 (6) | 10 (7) |
| FLHA_ECOLI | Flagellar biosynthesis protein flhA | flhA | IM | 9 (7) | 9 (8) | 11 (10) | 15 (12) | 11 (10) | 14 (13) |
| FLIC_ECOLI | Flagellin | fliC | OM | 473 (47) | 534 (44) | 552 (47) | 646 (46) | 497 (45) | 575 (48) |
| FLID_ECOLI | Flagellar hook-associated protein 2 | fliD | OM | 0 (0) | 5 (5) | 0 (0) | 2 (2) | 0 (0) | 2 (2) |
| FLIF_ECOLI | Flagellar M-ring protein | fliF | IM | 24 (19) | 28 (19) | 37 (25) | 42 (23) | 32 (22) | 45 (24) |
| FLIG_ECOLI | Flagellar motor switch protein FliG | fliG | p-IM | 0 (0) | 3 (3) | 3 (3) | 6 (6) | 4 (4) | 5 (4) |
| FLIL_ECOLI | Flagellar protein FliL | fliL | IM | 7 (5) | 10 (5) | 8 (4) | 10 (5) | 8 (5) | 9 (4) |
| FLIM_ECOLI | Flagellar motor switch protein FliM | fliM | p-IM | 4 (4) | 6 (6) | 3 (3) | 6 (6) | 5 (5) | 8 (8) |
| FLIP_ECOLI | Flagellar biosynthetic protein fliP | fliP | IM | 0 (0) | 2 (2) | 4 (4) | 5 (5) | 4 (4) | 3 (3) |
| FRDA_ECOLI | Fumarate reductase flavoprotein subunit | frdA | p-IM | 8 (8) | 5 (5) | 13 (13) | 10 (10) | 10 (10) | 7 (7) |
| FRDB_ECOLI | Fumarate reductase iron-sulfur subunit | frdB | p-IM | 0 (0) | 3 (2) | 4 (3) | 5 (4) | 5 (4) | 2 (2) |
| FRDC_ECOLI | Fumarate reductase subunit C | frdC | IM | 6 (3) | 5 (3) | 6 (3) | 6 (3) | 6 (3) | 3 (3) |
| FRDD_ECOLI | Fumarate reductase subunit D | frdD | IM | 4 (3) | 4 (3) | 3 (3) | 3 (3) | 2 (2) | 5 (3) |
| FTSA_ECOLI | Cell division protein FtsA | ftsA | p-IM | 9 (8) | 8 (8) | 7 (7) | 12 (11) | 8 (8) | 6 (6) |
| FTSB_ECOLI | Cell division protein FtsB | ftsB | IM | 1 (1) | 2 (2) | 1 (1) | 3 (2) | 1 (1) | 1 (1) |
| FTSE_ECOLI | Cell division ATP-binding protein FtsE | ftsE | p-IM | 3 (3) | 3 (3) | 1 (1) | 6 (6) | 1 (1) | 3 (3) |
| FTSI_ECOLI | Peptidoglycan synthetase ftsI | ftsI | IM | 13 (11) | 8 (7) | 8 (7) | 14 (13) | 8 (8) | 13 (12) |
| FTSK_ECOLI | DNA translocase FtsK | ftsK | IM | 0 (0) | 5 (4) | 0 (0) | 1 (1) | 1 (1) | 3 (3) |
| FTSL_ECOLI | Cell division protein FtsL | ftsL | IM | 2 (2) | 4 (4) | 2 (2) | 6 (4) | 1 (1) | 3 (2) |
| FTSN_ECOLI | Cell division protein ftsN | ftsN | IM | 4 (4) | 6 (6) | 4 (2) | 11 (7) | 5 (4) | 12 (7) |
| FTSQ_ECOLI | Cell division protein FtsQ | ftsQ | IM | 1 (1) | 0 (0) | 0 (0) | 1 (1) | 0 (0) | 0 (0) |
| FTSW_ECOLI | Putative lipid II flippase FtsW | ftsW | IM | 1 (1) | 2 (2) | 0 (0) | 0 (0) | 0 (0) | 0 (0) |
| FTSX_ECOLI | Cell division protein FtsX | ftsX | IM | 10 (9) | 8 (8) | 10 (9) | 8 (7) | 7 (7) | 10 (9) |
| FTSZ_ECOLI | Cell division protein FtsZ | ftsZ | p-IM | 20 (17) | 24 (19) | 17 (14) | 20 (15) | 18 (15) | 31 (22) |
| FUMA_ECOLI | Fumarate hydratase class I, aerobic | fumA | p-IM | 2 (2) | 0 (0) | 5 (5) | 0 (0) | 3 (3) | 0 (0) |
| G3P1_ECOLI | Glyceraldehyde-3-phosphate dehydrogenase A | gapA | p-IM | 2 (2) | 4 (3) | 17 (16) | 9 (5) | 12 (10) | 3 (3) |
| GLGA_ECOLI | Glycogen synthase | glgA | p-IM | 15 (13) | 22 (17) | 12 (11) | 17 (15) | 11 (11) | 15 (14) |
| GLMS_ECOLI | Glutamine--fructose-6-phosphate aminotransferase [isomerizing] | glmS | p-IM | 4 (4) | 0 (0) | 8 (8) | 0 (0) | 6 (6) | 3 (3) |
| GLNP_ECOLI | Glutamine transport system permease protein GlnP | glnP | IM | 4 (4) | 4 (4) | 4 (4) | 3 (3) | 4 (4) | 4 (4) |
| GLNQ_ECOLI | Glutamine transport ATP-binding protein glnQ | glnQ | p-IM | 14 (6) | 9 (5) | 13 (6) | 11 (4) | 10 (6) | 12 (6) |
| GLPA_ECOLI | Glycerol-3-phosphate dehydrogenase | glpA | p-IM | 1 (1) | 0 (0) | 0 (0) | 0 (0) | 0 (0) | 0 (0) |
| GLPF_ECOLI | Glycerol uptake facilitator protein | glpF | IM | 2 (1) | 3 (1) | 1 (1) | 4 (1) | 2 (1) | 0 (0) |
| GLPG_ECOLI | Rhomboid protease GlpG | glpG | IM | 2 (2) | 2 (2) | 1 (1) | 5 (4) | 2 (2) | 3 (3) |
| GLPK_ECOLI | Glycerol kinase | glpK | p-IM | 18 (18) | 29 (26) | 21 (19) | 22 (20) | 24 (22) | 27 (24) |
| GLYA_ECOLI | Serine hydroxymethyltransferase | glyA | p-IM | 6 (6) | 2 (2) | 10 (10) | 6 (6) | 7 (7) | 2 (2) |
| GRCA_ECOLI | Autonomous glycyl radical cofactor | grcA | p-IM | 3 (3) | 2 (2) | 4 (4) | 5 (5) | 5 (4) | 8 (7) |
| GSA_ECOLI | Glutamate-1-semialdehyde 2,1-aminomutase | hemL | p-IM | 8 (7) | 0 (0) | 10 (9) | 4 (4) | 6 (6) | 5 (5) |
| GUAA_ECOLI | GMP synthase [glutamine-hydrolyzing] | guaA | p-IM | 14 (7) | 9 (7) | 21 (12) | 10 (7) | 16 (14) | 14 (10) |
| GYRA_ECOLI | DNA gyrase subunit A | gyrA | p-IM | 3 (3) | 10 (10) | 8 (8) | 5 (5) | 5 (5) | 5 (5) |
| GYRB_ECOLI | DNA gyrase subunit B | gyrB | p-IM | 17 (14) | 12 (12) | 18 (17) | 21 (20) | 15 (13) | 16 (15) |
| HEMG_ECOLI | Protoporphyrinogen IX dehydrogenase [menaquinone] | hemG | p-IM | 4 (4) | 5 (5) | 4 (4) | 9 (7) | 3 (3) | 6 (6) |
| HEMX_ECOLI | Putative uroporphyrin-III C-methyltransferase | hemX | IM | 23 (17) | 35 (19) | 27 (19) | 35 (20) | 32 (23) | 26 (19) |
| HEMY_ECOLI | Protein HemY | hemY | IM | 6 (5) | 11 (9) | 12 (8) | 21 (13) | 9 (5) | 20 (13) |
| HFLC_ECOLI | Modulator of FtsH protease HflC | hflC | IM | 12 (9) | 21 (15) | 20 (14) | 28 (18) | 11 (10) | 25 (16) |
| HFLD_ECOLI | High frequency lysogenization protein HflD | hflD | p-IM | 1 (1) | 3 (3) | 1 (1) | 5 (5) | 1 (1) | 8 (7) |
| HFLK_ECOLI | HflK protein | hflK | IM | 34 (22) | 52 (26) | 47 (23) | 56 (27) | 42 (23) | 60 (26) |
| HNS_ECOLI | DNA-binding protein H-NS | hns | p-IM | 12 (5) | 6 (5) | 7 (4) | 1 (1) | 4 (3) | 4 (4) |
| HRPA_ECOLI | ATP-dependent helicase hrpA | hrpA | p-IM | 2 (2) | 9 (9) | 0 (0) | 10 (10) | 2 (2) | 9 (9) |
| HSLJ_ECOLI | Heat shock protein hslJ | hslJ | OM | 20 (14) | 20 (12) | 20 (14) | 20 (13) | 16 (13) | 15 (10) |
| HSLU_ECOLI | ATP-dependent protease ATPase subunit HslU | hslU | p-IM | 22 (18) | 19 (17) | 20 (17) | 21 (16) | 20 (17) | 21 (17) |
| HTPG_ECOLI | Chaperone protein HtpG | htpG | p-IM | 24 (22) | 11 (11) | 16 (15) | 11 (11) | 21 (20) | 26 (23) |
| HTPX_ECOLI | Protease HtpX | htpX | IM | 5 (5) | 7 (5) | 6 (6) | 9 (6) | 7 (6) | 6 (5) |
| IDH_ECOLI | Isocitrate dehydrogenase | icd | p-IM | 7 (7) | 4 (4) | 8 (8) | 5 (5) | 10 (9) | 5 (5) |
| IF2_ECOLI | Translation initiation factor IF-2 | infB | p-IM | 57 (33) | 34 (22) | 38 (28) | 37 (23) | 37 (25) | 40 (24) |
| IF3_ECOLI | Translation initiation factor IF-3 | infC | p-IM | 4 (4) | 4 (4) | 5 (4) | 5 (3) | 3 (3) | 4 (4) |
| IGAA_ECOLI | Putative membrane protein igaA homolog | yrfF | IM | 1 (1) | 4 (4) | 1 (1) | 4 (4) | 1 (1) | 3 (3) |
| IMDH_ECOLI | Inosine-5'-monophosphate dehydrogenase | guaB | p-IM | 19 (17) | 18 (16) | 27 (20) | 19 (18) | 31 (22) | 15 (13) |
| ISCS_ECOLI | Cysteine desulfurase IscS | iscS | p-IM | 4 (4) | 3 (3) | 3 (3) | 4 (4) | 3 (3) | 6 (6) |
| KDGL_ECOLI | Diacylglycerol kinase | dgkA | IM | 2 (2) | 5 (4) | 2 (2) | 3 (2) | 2 (2) | 3 (3) |
| KDPD_ECOLI | Histidine kinase | kdpD | IM | 6 (6) | 15 (12) | 6 (5) | 13 (12) | 7 (5) | 9 (8) |
| KDTA_ECOLI | 3-deoxy-D-manno-octulosonic acid transferase | waaA | IM | 1 (1) | 5 (5) | 4 (4) | 11 (11) | 5 (5) | 6 (6) |
| KPRS_ECOLI | Ribose-phosphate pyrophosphokinase | prs | p-IM | 11 (10) | 20 (11) | 18 (11) | 15 (9) | 10 (9) | 20 (12) |
| KPYK1_ECOLI | Pyruvate kinase | pykF | p-IM | 4 (4) | 1 (1) | 18 (17) | 8 (8) | 17 (15) | 2 (2) |
| KPYK2_ECOLI | Pyruvate kinase | pykA | p-IM | 17 (16) | 9 (9) | 21 (20) | 13 (13) | 17 (17) | 10 (10) |
| KUP_ECOLI | Low affinity potassium transport system protein kup | kup | IM | 4 (4) | 6 (5) | 2 (2) | 8 (7) | 4 (4) | 4 (4) |
| LACY_ECOLI | Lactose permease | lacY | IM | 4 (4) | 4 (4) | 4 (4) | 4 (4) | 4 (4) | 2 (2) |
| LAMB_ECOLI | Maltoporin | lamB | OM | 251 (35) | 221 (31) | 201 (28) | 188 (27) | 263 (32) | 197 (26) |
| LEP_ECOLI | Signal peptidase I | lepB | IM | 4 (4) | 7 (4) | 11 (8) | 18 (9) | 5 (5) | 16 (12) |
| LEPA_ECOLI | Elongation factor 4 | lepA | p-IM | 15 (14) | 15 (14) | 9 (9) | 11 (10) | 18 (12) | 15 (10) |
| LNT_ECOLI | Apolipoprotein N-acyltransferase | lnt | IM | 0 (0) | 4 (4) | 1 (1) | 6 (6) | 1 (1) | 4 (4) |
| LOIP_ECOLI | Putative metalloprotease yggG | yggG | OM | 9 (5) | 11 (8) | 6 (5) | 10 (6) | 11 (7) | 12 (7) |
| LOLB_ECOLI | Outer-membrane lipoprotein LolB | lolB | OM | 19 (16) | 21 (18) | 19 (18) | 21 (17) | 21 (15) | 18 (17) |
| LPOA_ECOLI | Penicillin-binding protein activator LpoA | lpoA | OM | 45 (29) | 40 (24) | 50 (29) | 30 (27) | 53 (30) | 45 (24) |
| LPTB_ECOLI | Lipopolysaccharide export system ATP-binding protein LptB | lptB | p-IM | 15 (14) | 14 (11) | 11 (9) | 16 (10) | 9 (8) | 7 (7) |
| LPTC_ECOLI | Lipopolysaccharide export system protein LptC | lptC | IM | 2 (2) | 1 (1) | 1 (1) | 1 (1) | 2 (2) | 2 (2) |
| LPTD_ECOLI | LPS-assembly protein LptD | lptD | OM | 98 (53) | 97 (44) | 106 (53) | 105 (52) | 108 (56) | 80 (44) |
| LPTE_ECOLI | LPS-assembly lipoprotein LptE | lptE | OM | 27 (15) | 33 (17) | 23 (16) | 33 (16) | 25 (15) | 27 (15) |
| LPTF_ECOLI | Lipopolysaccharide export system permease protein LptF | lptF | IM | 3 (3) | 5 (4) | 4 (4) | 5 (4) | 3 (3) | 4 (4) |
| LPTG_ECOLI | Lipopolysaccharide export system permease protein LptG | lptG | IM | 4 (4) | 6 (6) | 9 (8) | 6 (6) | 4 (4) | 4 (4) |
| MALF_ECOLI | Maltose transport system permease protein MalF | malF | IM | 9 (4) | 7 (4) | 10 (5) | 7 (6) | 10 (6) | 10 (7) |
| MALG_ECOLI | Maltose transport system permease protein MalG | malG | IM | 1 (1) | 1 (1) | 2 (2) | 4 (3) | 2 (2) | 1 (1) |
| MALK_ECOLI | Maltose/maltodextrin import ATP-binding protein MalK | malK | p-IM | 25 (19) | 24 (19) | 24 (17) | 25 (18) | 27 (21) | 26 (20) |
| MALM_ECOLI | Maltose operon P protein | malM | P | 0 (0) | 2 (2) | 0 (0) | 4 (4) | 2 (2) | 6 (5) |
| MALT_ECOLI | HTH-type transcriptional regulator MalT | malT | p-IM | 1 (1) | 5 (5) | 0 (0) | 7 (7) | 0 (0) | 4 (4) |
| MBHT_ECOLI | Hydrogenase-2 small chain | hybO | p-IM | 4 (3) | 2 (2) | 3 (2) | 2 (2) | 4 (3) | 1 (1) |
| MCP1_ECOLI | Methyl-accepting chemotaxis protein I | tsr | IM | 50 (29) | 47 (27) | 51 (30) | 63 (30) | 42 (31) | 71 (32) |
| MCP2_ECOLI | Methyl-accepting chemotaxis protein II | tar | IM | 39 (24) | 42 (26) | 35 (27) | 46 (25) | 35 (26) | 59 (30) |
| MDLA_ECOLI | Multidrug resistance-like ATP-binding protein mdlA | mdlA | IM | 0 (0) | 2 (2) | 1 (1) | 6 (6) | 2 (2) | 2 (2) |
| MDLB_ECOLI | Multidrug resistance-like ATP-binding protein MdlB | mdlB | IM | 1 (1) | 3 (3) | 1 (1) | 4 (4) | 2 (2) | 3 (3) |
| MDTA_ECOLI | Multidrug resistance protein MdtA | mdtA | p-IM | 0 (0) | 1 (1) | 1 (1) | 4 (4) | 0 (0) | 2 (2) |
| METK_ECOLI | S-adenosylmethionine synthase | metK | p-IM | 5 (5) | 5 (5) | 7 (7) | 6 (6) | 5 (5) | 8 (8) |
| METN_ECOLI | Methionine import ATP-binding protein MetN | metN | p-IM | 0 (0) | 3 (3) | 0 (0) | 6 (6) | 1 (1) | 2 (2) |
| MGLA_ECOLI | Galactose/methyl galactoside import ATP-binding protein MglA | mglA | p-IM | 12 (12) | 14 (11) | 8 (7) | 14 (13) | 14 (12) | 13 (13) |
| MIND_ECOLI | Septum site-determining protein MinD | minD | p-IM | 15 (13) | 16 (12) | 19 (15) | 17 (13) | 13 (10) | 15 (12) |
| MINE_ECOLI | Cell division topological specificity factor | minE | p-IM | 6 (5) | 3 (3) | 1 (1) | 1 (1) | 2 (2) | 2 (2) |
| MLAF_ECOLI | Probable phospholipid import ATP-binding protein MlaF | mlaF | p-IM | 1 (1) | 0 (0) | 1 (1) | 2 (2) | 0 (0) | 0 (0) |
| MLTA_ECOLI | Membrane-bound lytic murein transglycosylase A | mltA | OM | 13 (10) | 17 (11) | 16 (14) | 19 (11) | 21 (13) | 12 (9) |
| MLTB_ECOLI | Membrane-bound lytic murein transglycosylase B | mltB | OM | 6 (5) | 8 (7) | 8 (7) | 6 (5) | 7 (6) | 5 (5) |
| MLTC_ECOLI | Membrane-bound lytic murein transglycosylase C | mltC | OM | 1 (1) | 5 (4) | 2 (1) | 1 (1) | 2 (1) | 4 (3) |
| MNMG_ECOLI | tRNA uridine 5-carboxymethylaminomethyl modification enzyme MnmG | mnmG | p-IM | 4 (4) | 0 (0) | 1 (1) | 0 (0) | 1 (1) | 0 (0) |
| MOTA_ECOLI | Chemotaxis motA protein | motA | IM | 2 (2) | 2 (2) | 2 (2) | 4 (3) | 2 (2) | 1 (1) |
| MOTB_ECOLI | Chemotaxis motB protein | motB | IM | 1 (1) | 1 (1) | 1 (1) | 3 (3) | 0 (0) | 4 (4) |
| MRAY_ECOLI | Phospho-N-acetylmuramoyl-pentapeptide-transferase | mraY | IM | 2 (2) | 5 (3) | 3 (2) | 7 (4) | 2 (2) | 2 (2) |
| MREC_ECOLI | Rod shape-determining protein mreC | mreC | IM | 0 (0) | 3 (2) | 0 (0) | 6 (4) | 2 (2) | 2 (2) |
| MSBA_ECOLI | Lipid A export ATP-binding/permease protein MsbA | msbA | IM | 17 (16) | 27 (17) | 17 (14) | 32 (25) | 23 (18) | 22 (19) |
| MSCL_ECOLI | Large-conductance mechanosensitive channel | mscL | IM | 7 (4) | 8 (4) | 5 (3) | 10 (5) | 8 (3) | 7 (6) |
| MUKF_ECOLI | Chromosome partition protein MukF | mukF | p-IM | 3 (3) | 2 (2) | 2 (2) | 1 (1) | 1 (1) | 4 (4) |
| MURC_ECOLI | UDP-N-acetylmuramate--L-alanine ligase | murC | p-IM | 5 (5) | 1 (1) | 3 (3) | 2 (2) | 3 (3) | 5 (4) |
| NAPA_ECOLI | P nitrate reductase | napA | P | 0 (0) | 0 (0) | 1 (1) | 6 (6) | 2 (2) | 0 (0) |
| NAPC_ECOLI | Cytochrome c-type protein NapC | napC | IM | 1 (1) | 1 (1) | 0 (0) | 1 (1) | 2 (1) | 1 (1) |
| NARG_ECOLI | Respiratory nitrate reductase 1 alpha chain | narG | p-IM | 3 (3) | 3 (3) | 5 (5) | 1 (1) | 2 (2) | 0 (0) |
| NDK_ECOLI | Nucleoside diphosphate kinase | ndk | p-IM | 4 (4) | 1 (1) | 3 (3) | 1 (1) | 3 (3) | 2 (2) |
| NFRA_ECOLI | Bacteriophage N4 adsorption protein A | nfrA | OM | 17 (15) | 24 (23) | 15 (15) | 8 (8) | 19 (19) | 6 (6) |
| NHAA_ECOLI | Na(+)/H(+) antiporter NhaA | nhaA | IM | 2 (2) | 2 (2) | 3 (2) | 3 (2) | 4 (4) | 2 (2) |
| NHAB_ECOLI | Na(+)/H(+) antiporter NhaB | nhaB | IM | 8 (6) | 6 (5) | 6 (6) | 6 (5) | 4 (4) | 6 (6) |
| NLPA_ECOLI | Lipoprotein | nlpA | IM | 5 (5) | 3 (3) | 4 (4) | 5 (5) | 4 (4) | 4 (4) |
| NLPD_ECOLI | Lipoprotein nlpD | nlpD | OM | 21 (10) | 26 (11) | 28 (11) | 26 (11) | 29 (13) | 21 (11) |
| NLPI_ECOLI | Lipoprotein NlpI | nlpI | OM | 12 (12) | 12 (11) | 14 (11) | 7 (7) | 11 (10) | 11 (9) |
| NMPC_ECOLI | Outer membrane porin protein nmpC | nmpC | OM | 352 (23) | 419 (24) | 340 (27) | 371 (27) | 421 (24) | 291 (22) |
| NUOA_ECOLI | NADH-quinone oxidoreductase subunit A | nuoA | IM | 8 (4) | 8 (5) | 5 (4) | 8 (5) | 9 (4) | 8 (4) |
| NUOB_ECOLI | NADH-quinone oxidoreductase subunit B | nuoB | p-IM | 2 (2) | 7 (5) | 0 (0) | 2 (2) | 3 (3) | 5 (3) |
| NUOCD_ECOLI | NADH-quinone oxidoreductase subunit C/D | nuoC | p-IM | 15 (14) | 23 (20) | 15 (14) | 27 (23) | 14 (13) | 27 (20) |
| NUOF_ECOLI | NADH dehydrogenase I chain F | nuoF | p-IM | 14 (14) | 25 (21) | 6 (6) | 22 (20) | 6 (6) | 14 (14) |
| NUOG_ECOLI | NADH-quinone oxidoreductase subunit G | nuoG | p-IM | 3 (3) | 16 (14) | 15 (14) | 24 (18) | 12 (10) | 14 (12) |
| NUOH_ECOLI | NADH-quinone oxidoreductase subunit H | nuoH | IM | 7 (5) | 9 (6) | 5 (4) | 8 (5) | 4 (2) | 9 (5) |
| NUOI_ECOLI | NADH-quinone oxidoreductase subunit I | nuoI | p-IM | 8 (8) | 11 (10) | 7 (7) | 19 (13) | 8 (8) | 15 (11) |
| NUOJ_ECOLI | NADH-quinone oxidoreductase subunit J | nuoJ | IM | 3 (1) | 4 (1) | 1 (1) | 4 (1) | 2 (1) | 3 (1) |
| NUOK_ECOLI | NADH-quinone oxidoreductase subunit K | nuoK | IM | 4 (4) | 5 (4) | 4 (4) | 7 (6) | 4 (4) | 4 (3) |
| NUOL_ECOLI | NADH dehydrogenase I chain L | nuoL | IM | 2 (2) | 5 (2) | 2 (2) | 5 (3) | 2 (2) | 5 (4) |
| NUPC_ECOLI | Nucleoside permease NupC | nupC | IM | 10 (8) | 13 (10) | 12 (9) | 10 (6) | 10 (9) | 10 (8) |
| NUSA_ECOLI | Transcription termination/antitermination protein NusA | nusA | p-IM | 6 (5) | 0 (0) | 7 (7) | 0 (0) | 5 (5) | 1 (1) |
| NUSG_ECOLI | Transcription termination/antitermination protein NusG | nusG | p-IM | 6 (6) | 4 (4) | 3 (3) | 2 (2) | 0 (0) | 4 (4) |
| ODO1_ECOLI | 2-oxoglutarate dehydrogenase E1 component | sucA | p-IM | 36 (23) | 35 (26) | 22 (21) | 34 (26) | 32 (29) | 44 (29) |
| ODO2_ECOLI | Dihydrolipoyllysine-residue succinyltransferase component of 2-oxoglutarate dehydrogenase complex | sucB | p-IM | 21 (16) | 36 (19) | 22 (16) | 28 (17) | 26 (20) | 25 (18) |
| ODP1_ECOLI | Pyruvate dehydrogenase E1 component | aceE | p-IM | 62 (50) | 77 (40) | 88 (48) | 78 (45) | 63 (43) | 59 (38) |
| OMPA_ECOLI | Outer membrane protein A | ompA | OM | 434 (31) | 534 (33) | 516 (38) | 494 (32) | 469 (33) | 397 (35) |
| OMPC_ECOLI | Outer membrane protein C | ompC | OM | 320 (22) | 448 (25) | 289 (27) | 366 (24) | 336 (23) | 292 (25) |
| OMPF_ECOLI | Outer membrane protein F | ompF | OM | 78 (21) | 100 (22) | 92 (23) | 112 (21) | 95 (21) | 71 (22) |
| OMPR_ECOLI | Transcriptional regulatory protein OmpR | ompR | p-IM | 4 (4) | 7 (7) | 3 (3) | 4 (4) | 2 (2) | 5 (5) |
| OMPT_ECOLI | Protease VII | ompT | OM | 22 (14) | 20 (17) | 19 (13) | 16 (14) | 21 (17) | 19 (17) |
| OMPX_ECOLI | Outer membrane protein X | ompX | OM | 77 (11) | 78 (11) | 55 (10) | 56 (10) | 66 (10) | 51 (10) |
| OSME_ECOLI | Osmotically inducible lipoprotein E | osmE | OM | 23 (6) | 19 (5) | 23 (6) | 18 (6) | 23 (6) | 10 (5) |
| PA1_ECOLI | Phospholipase A1 | pldA | OM | 14 (9) | 16 (10) | 10 (8) | 12 (9) | 14 (9) | 12 (9) |
| PAL_ECOLI | Peptidoglycan-associated lipoprotein | pal | OM | 59 (20) | 57 (18) | 49 (19) | 41 (15) | 53 (23) | 55 (22) |
| PARC_ECOLI | DNA topoisomerase 4 subunit A | parC | p-IM | 2 (2) | 4 (4) | 1 (1) | 3 (3) | 0 (0) | 4 (4) |
| PBP2_ECOLI | Penicillin-binding protein 2 | mrdA | IM | 4 (4) | 4 (4) | 2 (2) | 8 (8) | 4 (4) | 10 (10) |
| PBPA_ECOLI | Penicillin-binding protein 1A | mrcA | IM | 18 (17) | 20 (16) | 19 (18) | 40 (32) | 19 (18) | 26 (23) |
| PBPB_ECOLI | Penicillin-binding protein 1B | mrcB | IM | 20 (20) | 26 (21) | 24 (24) | 29 (26) | 22 (21) | 24 (22) |
| PDXH_ECOLI | Pyridoxine/pyridoxamine 5'-phosphate oxidase | pdxH | p-IM | 0 (0) | 4 (4) | 0 (0) | 1 (1) | 2 (2) | 0 (0) |
| PGK_ECOLI | Phosphoglycerate kinase | pgk | p-IM | 0 (0) | 1 (1) | 4 (4) | 0 (0) | 1 (1) | 1 (1) |
| PGSA_ECOLI | CDP-diacylglycerol--glycerol-3-phosphate 3-phosphatidyltransferase | pgsA | IM | 1 (1) | 3 (3) | 1 (1) | 3 (3) | 1 (1) | 3 (3) |
| PHOU_ECOLI | Phosphate-specific transport system accessory protein PhoU | phoU | p-IM | 1 (1) | 4 (4) | 2 (2) | 1 (1) | 0 (0) | 0 (0) |
| PHSM_ECOLI | Alpha-1,4 glucan phosphorylase | malP | IM | 40 (35) | 36 (27) | 45 (39) | 41 (35) | 61 (34) | 35 (25) |
| PITA_ECOLI | Low-affinity inorganic phosphate transporter 1 | pitA | IM | 9 (9) | 9 (7) | 8 (8) | 12 (11) | 7 (7) | 6 (6) |
| PLSB_ECOLI | Glycerol-3-phosphate acyltransferase | plsB | p-IM | 26 (23) | 26 (22) | 30 (27) | 43 (32) | 33 (23) | 35 (23) |
| PLSC_ECOLI | 1-acyl-sn-glycerol-3-phosphate acyltransferase | plsC | IM | 1 (1) | 2 (2) | 3 (3) | 7 (4) | 3 (3) | 1 (1) |
| PNP_ECOLI | Polyribonucleotide nucleotidyltransferase | pnp | p-IM | 25 (23) | 18 (16) | 25 (22) | 17 (17) | 28 (24) | 22 (20) |
| PNTA_ECOLI | NAD(P) transhydrogenase subunit alpha | pntA | IM | 29 (16) | 31 (16) | 32 (17) | 37 (17) | 29 (17) | 36 (16) |
| PNTB_ECOLI | NAD(P) transhydrogenase subunit beta | pntB | IM | 13 (10) | 26 (13) | 16 (13) | 25 (13) | 14 (11) | 28 (14) |
| POTA_ECOLI | Spermidine/putrescine import ATP-binding protein PotA | potA | p-IM | 4 (4) | 6 (6) | 3 (3) | 4 (4) | 3 (2) | 3 (3) |
| POTB_ECOLI | Spermidine/putrescine transport system permease protein potB | potB | IM | 2 (2) | 2 (2) | 0 (0) | 3 (3) | 1 (1) | 4 (3) |
| PPID_ECOLI | Peptidyl-prolyl cis-trans isomerase D | ppiD | IM | 40 (32) | 34 (27) | 41 (33) | 43 (31) | 39 (32) | 42 (33) |
| PPSA_ECOLI | Phosphoenolpyruvate synthase | ppsA | p-IM | 24 (22) | 22 (18) | 17 (16) | 25 (21) | 25 (21) | 35 (29) |
| PPX_ECOLI | Exopolyphosphatase | ppx | p-IM | 3 (3) | 3 (3) | 0 (0) | 3 (3) | 1 (1) | 3 (3) |
| PQIB_ECOLI | Paraquat-inducible protein B | pqiB | IM | 24 (23) | 21 (19) | 27 (24) | 27 (24) | 23 (22) | 25 (23) |
| PRC_ECOLI | Tail-specific protease | prc | p-IM | 5 (5) | 8 (8) | 5 (5) | 23 (19) | 8 (8) | 12 (11) |
| PROP_ECOLI | Proline/betaine transporter | proP | IM | 2 (2) | 0 (0) | 1 (1) | 4 (4) | 0 (0) | 2 (2) |
| PROQ_ECOLI | RNA chaperone ProQ | proQ | p-IM | 4 (4) | 7 (7) | 8 (7) | 4 (4) | 4 (4) | 2 (2) |
| PSD_ECOLI | Phosphatidylserine decarboxylase proenzyme | psd | p-IM | 7 (7) | 7 (6) | 9 (7) | 10 (8) | 10 (8) | 9 (8) |
| PSPB_ECOLI | Phage shock protein B | pspB | IM | 1 (1) | 3 (3) | 1 (1) | 4 (3) | 1 (1) | 5 (4) |
| PSTB_ECOLI | Phosphate import ATP-binding protein PstB | pstB | p-IM | 4 (4) | 9 (8) | 5 (5) | 5 (5) | 4 (4) | 5 (5) |
| PT1_ECOLI | Phosphoenolpyruvate-protein phosphotransferase | ptsI | p-IM | 15 (13) | 9 (9) | 10 (9) | 4 (4) | 9 (9) | 14 (13) |
| PTA_ECOLI | Phosphate acetyltransferase | pta | P | 35 (24) | 20 (15) | 33 (23) | 22 (19) | 29 (23) | 24 (21) |
| PTFBC_ECOLI | PTS system, fructose-specific IIBC component | fruA | IM | 15 (8) | 13 (7) | 12 (7) | 16 (7) | 12 (7) | 12 (6) |
| PTGCB_ECOLI | PTS system glucose-specific EIICB component | ptsG | IM | 35 (18) | 48 (20) | 40 (19) | 58 (20) | 41 (17) | 44 (16) |
| PTM3C_ECOLI | PTS system, mannitol-specific IIABC component | mtlA | IM | 8 (8) | 12 (7) | 8 (8) | 10 (8) | 7 (7) | 8 (8) |
| PTNAB_ECOLI | PTS system mannose-specific EIIAB component | manX | p-IM | 9 (9) | 5 (5) | 15 (14) | 12 (12) | 10 (10) | 11 (10) |
| PTNC_ECOLI | Mannose permease IIC component | manY | IM | 3 (2) | 3 (2) | 3 (2) | 4 (2) | 5 (2) | 4 (2) |
| PTND_ECOLI | Mannose permease IID component | manZ | IM | 16 (10) | 11 (8) | 17 (10) | 17 (12) | 12 (9) | 10 (8) |
| PTTBC_ECOLI | PTS system, trehalose-specific IIBC component | treB | IM | 11 (6) | 12 (6) | 10 (7) | 14 (8) | 11 (7) | 8 (6) |
| PTW3C_ECOLI | PTS system, N-acetylglucosamine-specific IIABC component | nagE | IM | 12 (8) | 11 (7) | 8 (7) | 18 (10) | 12 (9) | 13 (7) |
| PUR7_ECOLI | Phosphoribosylaminoimidazole-succinocarboxamide synthase | purC | p-IM | 8 (7) | 5 (4) | 5 (5) | 2 (2) | 4 (4) | 8 (8) |
| PUR8_ECOLI | Adenylosuccinate lyase | purB | p-IM | 20 (19) | 22 (17) | 21 (18) | 21 (17) | 17 (14) | 21 (19) |
| PURA_ECOLI | Adenylosuccinate synthetase | purA | p-IM | 17 (11) | 6 (5) | 15 (9) | 11 (9) | 12 (10) | 12 (8) |
| PUTA_ECOLI | PutA protein | putA | p-IM | 37 (33) | 44 (35) | 24 (24) | 52 (41) | 28 (26) | 38 (33) |
| PUTP_ECOLI | Sodium/proline symporter | putP | IM | 0 (0) | 6 (5) | 4 (4) | 6 (5) | 2 (2) | 6 (5) |
| PYRD_ECOLI | Dihydroorotate dehydrogenase (quinone) | pyrD | p-IM | 4 (3) | 4 (3) | 7 (6) | 16 (12) | 4 (3) | 17 (15) |
| PYRG_ECOLI | CTP synthase | pyrG | p-IM | 25 (24) | 10 (9) | 16 (15) | 9 (9) | 18 (16) | 14 (13) |
| PYRH_ECOLI | Uridylate kinase | pyrH | p-IM | 1 (1) | 0 (0) | 0 (0) | 0 (0) | 0 (0) | 0 (0) |
| QMCA_ECOLI | Protein QmcA | qmcA | IM | 0 (0) | 2 (2) | 3 (3) | 6 (6) | 0 (0) | 3 (3) |
| RBFA_ECOLI | Ribosome-binding factor A | rbfA | p-IM | 5 (5) | 5 (5) | 0 (0) | 4 (3) | 1 (1) | 4 (4) |
| RCSB_ECOLI | Transcriptional regulatory protein RcsB | rcsB | p-IM | 3 (3) | 3 (3) | 1 (1) | 1 (1) | 2 (2) | 3 (3) |
| RCSF_ECOLI | OM RcsF | rcsF | OM | 15 (7) | 17 (8) | 12 (6) | 12 (6) | 16 (7) | 19 (7) |
| RECA_ECOLI | Protein RecA | recA | p-IM | 12 (9) | 17 (8) | 9 (6) | 22 (10) | 12 (8) | 20 (11) |
| RELA_ECOLI | GTP pyrophosphokinase | relA | p-IM | 1 (1) | 6 (6) | 1 (1) | 6 (6) | 1 (1) | 6 (5) |
| RFAG_ECOLI | Lipopolysaccharide core biosynthesis protein rfaG | rfaG | p-IM | 1 (1) | 4 (4) | 4 (4) | 11 (10) | 2 (2) | 7 (7) |
| RFAP_ECOLI | Lipopolysaccharide core heptose(I) kinase | rfaP | p-IM | 1 (1) | 2 (2) | 1 (1) | 6 (6) | 1 (1) | 2 (2) |
| RHLB_ECOLI | ATP-dependent RNA helicase RhlB | rhlB | p-IM | 4 (4) | 6 (6) | 3 (3) | 6 (6) | 5 (5) | 7 (6) |
| RHO_ECOLI | Transcription termination factor Rho | rho | p-IM | 32 (21) | 32 (19) | 41 (25) | 34 (21) | 34 (23) | 33 (22) |
| RIR1_ECOLI | Ribonucleoside-diphosphate reductase | nrdA | p-IM | 29 (29) | 18 (17) | 25 (22) | 19 (17) | 23 (23) | 19 (19) |
| RLME_ECOLI | Ribosomal RNA large subunit methyltransferase E | rlmE | p-IM | 1 (1) | 2 (2) | 2 (2) | 4 (4) | 0 (0) | 4 (4) |
| RLPA_ECOLI | Rare lipoprotein A | rlpA | OM | 20 (8) | 18 (10) | 20 (12) | 13 (8) | 20 (11) | 17 (10) |
| RNC_ECOLI | Ribonuclease 3 | rnc | v | 3 (3) | 2 (2) | 2 (2) | 0 (0) | 5 (5) | 0 (0) |
| RNE_ECOLI | Ribonuclease E | rne | p-IM | 29 (25) | 42 (18) | 15 (12) | 44 (21) | 26 (21) | 21 (15) |
| RODA_ECOLI | Rod shape-determining protein rodA | mrdB | IM | 2 (2) | 3 (3) | 4 (4) | 2 (2) | 0 (0) | 4 (4) |
| RODZ_ECOLI | Cytoskeleton protein RodZ | rodZ | IM | 10 (7) | 21 (11) | 19 (10) | 21 (11) | 18 (11) | 18 (9) |
| RPOA_ECOLI | DNA-directed RNA polymerase subunit alpha | rpoA | p-IM | 9 (8) | 7 (7) | 12 (11) | 6 (6) | 6 (6) | 10 (10) |
| RPOB_ECOLI | DNA-directed RNA polymerase subunit beta | rpoB | p-IM | 79 (68) | 42 (35) | 62 (57) | 54 (48) | 68 (62) | 55 (47) |
| RPOC_ECOLI | DNA-directed RNA polymerase subunit beta' | rpoC | p-IM | 92 (77) | 129 (63) | 71 (64) | 118 (69) | 99 (73) | 101 (70) |
| RPOD_ECOLI | RNA polymerase sigma factor RpoD | rpoD | p-IM | 7 (6) | 11 (9) | 7 (6) | 11 (9) | 8 (7) | 13 (8) |
| RPOE_ECOLI | ECF RNA polymerase sigma-E factor | rpoE | p-IM | 0 (0) | 2 (2) | 0 (0) | 3 (3) | 0 (0) | 5 (5) |
| SDAC_ECOLI | Serine transporter | sdaC | IM | 14 (11) | 19 (11) | 14 (9) | 20 (11) | 16 (10) | 18 (10) |
| SDHL_ECOLI | L-serine dehydratase 1 | sdaA | p-IM | 6 (6) | 6 (5) | 6 (5) | 3 (3) | 5 (5) | 5 (4) |
| SECA_ECOLI | Protein translocase subunit SecA | secA | p-IM | 45 (24) | 62 (47) | 48 (41) | 111 (61) | 49 (36) | 101 (56) |
| SECD_ECOLI | Protein translocase subunit SecD | secD | IM | 56 (28) | 54 (26) | 57 (30) | 67 (33) | 62 (30) | 60 (31) |
| SECE_ECOLI | Protein translocase subunit SecE | secE | IM | 5 (3) | 6 (3) | 5 (3) | 4 (4) | 4 (3) | 3 (2) |
| SECF_ECOLI | Protein translocase subunit SecF | secF | IM | 9 (6) | 14 (9) | 9 (4) | 15 (6) | 13 (9) | 8 (5) |
| SECG_ECOLI | Protein-export membrane protein SecG | secG | IM | 7 (3) | 6 (2) | 4 (2) | 6 (2) | 6 (2) | 6 (2) |
| SECY_ECOLI | Protein translocase subunit SecY | secY | IM | 16 (8) | 18 (9) | 15 (9) | 21 (8) | 13 (6) | 18 (9) |
| SEQA_ECOLI | Negative modulator of initiation of replication | seqA | p-IM | 4 (4) | 6 (5) | 4 (4) | 7 (5) | 5 (5) | 6 (5) |
| SKP_ECOLI | Chaperone protein Skp | skp | P | 9 (6) | 13 (6) | 11 (8) | 9 (6) | 8 (5) | 13 (7) |
| SLP_ECOLI | Outer membrane protein slp | slp | OM | 5 (5) | 4 (4) | 4 (4) | 2 (2) | 2 (2) | 5 (4) |
| SLYB_ECOLI | OM slyB | slyB | OM | 38 (11) | 40 (11) | 38 (12) | 34 (10) | 36 (11) | 23 (8) |
| SOHB_ECOLI | Possible protease sohB | sohB | IM | 1 (1) | 3 (3) | 3 (3) | 9 (7) | 5 (4) | 11 (8) |
| SPPA_ECOLI | Protease 4 | sppA | IM | 6 (6) | 7 (6) | 6 (6) | 7 (6) | 7 (7) | 12 (7) |
| SRMB_ECOLI | ATP-dependent RNA helicase SrmB | srmB | p-IM | 6 (6) | 8 (8) | 5 (5) | 4 (4) | 4 (4) | 2 (2) |
| STHA_ECOLI | Soluble pyridine nucleotide transhydrogenase | sthA | p-IM | 5 (5) | 6 (6) | 3 (3) | 5 (5) | 8 (8) | 8 (8) |
| STPA_ECOLI | DNA-binding protein | stpA | p-IM | 8 (6) | 8 (5) | 3 (3) | 1 (1) | 3 (3) | 3 (2) |
| SUCC_ECOLI | Succinyl-CoA ligase [ADP-forming] subunit beta | sucC | p-IM | 3 (3) | 4 (4) | 7 (7) | 5 (5) | 6 (6) | 4 (4) |
| SUHB_ECOLI | Inositol-1-monophosphatase | suhB | p-IM | 5 (4) | 8 (7) | 5 (5) | 4 (4) | 4 (4) | 4 (4) |
| SYFB_ECOLI | Phenylalanine--tRNA ligase beta subunit | pheT | p-IM | 1 (1) | 3 (3) | 9 (9) | 1 (1) | 6 (6) | 0 (0) |
| SYGB_ECOLI | Glycine--tRNA ligase beta subunit | glyS | p-IM | 10 (9) | 10 (10) | 15 (15) | 1 (1) | 11 (11) | 16 (15) |
| SYM_ECOLI | Methionine--tRNA ligase | metG | p-IM | 7 (7) | 9 (9) | 10 (10) | 1 (1) | 11 (11) | 6 (6) |
| SYN_ECOLI | Asparagine--tRNA ligase | asnS | p-IM | 20 (18) | 17 (15) | 15 (13) | 9 (8) | 18 (17) | 14 (13) |
| SYP_ECOLI | Proline--tRNA ligase | proS | p-IM | 14 (14) | 9 (9) | 15 (15) | 4 (4) | 12 (12) | 9 (9) |
| SYT_ECOLI | Threonine--tRNA ligase | thrS | p-IM | 5 (5) | 5 (5) | 6 (6) | 0 (0) | 7 (7) | 5 (5) |
| SYY_ECOLI | Tyrosine--tRNA ligase | tyrS | p-IM | 3 (2) | 2 (1) | 7 (6) | 7 (6) | 6 (5) | 6 (6) |
| TALB_ECOLI | Transaldolase B | talB | p-IM | 1 (1) | 0 (0) | 5 (5) | 0 (0) | 4 (4) | 3 (3) |
| TATA_ECOLI | Sec-independent protein translocase protein TatA | tatA | IM | 6 (3) | 7 (3) | 6 (3) | 6 (3) | 6 (3) | 7 (4) |
| TATB_ECOLI | Sec-independent protein translocase protein TatB | tatB | IM | 7 (6) | 6 (6) | 4 (4) | 7 (6) | 5 (5) | 5 (5) |
| TATC_ECOLI | Sec-independent protein translocase protein TatC | tatC | IM | 0 (0) | 1 (1) | 0 (0) | 4 (2) | 0 (0) | 1 (1) |
| TATE_ECOLI | Probable Sec-independent protein translocase protein TatE | tatE | IM | 3 (2) | 4 (3) | 3 (2) | 4 (3) | 3 (2) | 2 (2) |
| TDCB_ECOLI | L-threonine dehydratase catabolic TdcB | tdcB | p-IM | 4 (4) | 0 (0) | 5 (5) | 0 (0) | 4 (4) | 0 (0) |
| TDCC_ECOLI | Threonine/serine transporter TdcC | tdcC | IM | 3 (2) | 4 (3) | 5 (4) | 5 (3) | 5 (2) | 4 (4) |
| TIG_ECOLI | Trigger factor | tig | p-IM | 21 (18) | 18 (17) | 24 (22) | 18 (15) | 25 (23) | 21 (19) |
| TOLA_ECOLI | TolA protein | tolA | IM | 4 (4) | 6 (5) | 4 (4) | 7 (6) | 4 (4) | 5 (5) |
| TOLC_ECOLI | Outer membrane protein tolC | tolC | OM | 96 (25) | 110 (28) | 88 (26) | 89 (25) | 103 (29) | 79 (26) |
| TOLQ_ECOLI | TolQ protein | tolQ | IM | 6 (5) | 8 (6) | 7 (6) | 8 (7) | 7 (6) | 7 (6) |
| TOLR_ECOLI | Protein TolR | tolR | IM | 3 (3) | 7 (3) | 3 (3) | 3 (3) | 2 (2) | 5 (3) |
| TRMJ_ECOLI | tRNA (cytidine/uridine-2'-O-)-methyltransferase TrmJ | trmJ | p-IM | 6 (6) | 1 (1) | 3 (3) | 4 (4) | 2 (2) | 2 (2) |
| TSX_ECOLI | Nucleoside-specific channel-forming protein tsx | tsx | OM | 30 (10) | 37 (12) | 27 (7) | 25 (10) | 31 (10) | 27 (9) |
| TYPA_ECOLI | GTP-binding protein TypA/BipA | typA | p-IM | 16 (13) | 11 (10) | 14 (9) | 12 (11) | 16 (11) | 13 (9) |
| UBIB_ECOLI | Probable protein kinase UbiB | ubiB | IM | 5 (5) | 8 (8) | 6 (6) | 10 (10) | 5 (5) | 9 (9) |
| UPPP_ECOLI | Undecaprenyl-diphosphatase | uppP | IM | 1 (1) | 3 (3) | 1 (1) | 3 (3) | 0 (0) | 3 (3) |
| USPA_ECOLI | Universal stress protein A | uspA | p-IM | 3 (2) | 0 (0) | 2 (2) | 0 (0) | 1 (1) | 1 (1) |
| UVRA_ECOLI | UvrABC system protein A | uvrA | p-IM | 1 (1) | 5 (5) | 1 (1) | 3 (3) | 2 (2) | 4 (4) |
| WECA_ECOLI | Undecaprenyl-phosphate alpha-N-acetylglucosaminyl 1-phosphate transferase | wecA | IM | 2 (2) | 2 (2) | 3 (3) | 4 (2) | 0 (0) | 3 (2) |
| WZZB_ECOLI | Chain length determinant protein | wzzB | IM | 7 (6) | 8 (7) | 6 (6) | 10 (8) | 7 (5) | 14 (12) |
| WZZE_ECOLI | Lipopolysaccharide biosynthesis protein wzzE | wzzE | IM | 0 (0) | 2 (2) | 0 (0) | 4 (4) | 1 (1) | 7 (7) |
| YADG_ECOLI | Hypothetical ABC transporter ATP-binding protein yadG | yadG | p-IM | 0 (0) | 0 (0) | 3 (3) | 3 (3) | 0 (0) | 2 (2) |
| YAGU_ECOLI | Uncharacterized protein | yagU | IM | 6 (5) | 8 (5) | 8 (7) | 10 (8) | 12 (7) | 8 (6) |
| YAIW_ECOLI | Uncharacterized protein | yaiW | OM | 10 (9) | 9 (8) | 11 (10) | 7 (6) | 10 (9) | 9 (8) |
| YAJC_ECOLI | UPF0092 membrane protein YajC | yajC | IM | 12 (8) | 8 (6) | 8 (6) | 12 (8) | 14 (11) | 7 (6) |
| YAJG_ECOLI | Uncharacterized lipoprotein YajG | yajG | OM | 8 (5) | 9 (5) | 8 (5) | 6 (4) | 7 (5) | 5 (3) |
| YBAL_ECOLI | Uncharacterized protein | ybaL | IM | 2 (2) | 4 (4) | 4 (4) | 7 (6) | 2 (2) | 4 (4) |
| YBBO_ECOLI | Uncharacterized oxidoreductase YbbO | ybbO | p-IM | 1 (1) | 2 (2) | 1 (1) | 2 (2) | 0 (0) | 3 (3) |
| YBHC_ECOLI | Putative lipoprotein ybHC | ybhC | OM | 43 (20) | 38 (17) | 36 (16) | 36 (18) | 47 (19) | 31 (16) |
| YBHG_ECOLI | UPF0194 membrane protein YbhG | ybhG | IM | 0 (0) | 0 (0) | 0 (0) | 4 (3) | 1 (1) | 2 (2) |
| YBIS_ECOLI | Probable L,D-transpeptidase YbiS | ybiS | OM | 2 (2) | 4 (4) | 5 (5) | 3 (3) | 2 (2) | 2 (2) |
| YBIT_ECOLI | Uncharacterized ABC transporter ATP-binding protein YbiT | ybiT | p-IM | 8 (7) | 0 (0) | 4 (4) | 0 (0) | 5 (5) | 4 (4) |
| YBJP_ECOLI | Putative lipoprotein ybjP | ybjP | OM | 11 (6) | 15 (6) | 9 (6) | 11 (6) | 13 (6) | 14 (6) |
| YCCF_ECOLI | Uncharacterized protein | yccF | IM | 4 (2) | 6 (4) | 4 (2) | 7 (4) | 4 (2) | 4 (4) |
| YCEG_ECOLI | Uncharacterized protein | yceG | IM | 0 (0) | 1 (1) | 3 (2) | 6 (4) | 1 (1) | 2 (1) |
| YCFL_ECOLI | Uncharacterized protein | ycfL | OM | 4 (3) | 3 (3) | 4 (3) | 1 (1) | 2 (2) | 2 (2) |
| YCGR_ECOLI | Flagellar brake protein YcgR | ycgR | p-IM | 4 (4) | 9 (7) | 2 (2) | 6 (5) | 2 (2) | 4 (4) |
| YDBH_ECOLI | Uncharacterized protein | ydbH | IM | 4 (4) | 6 (4) | 1 (1) | 3 (3) | 6 (6) | 2 (2) |
| YDBJ_ECOLI | Uncharacterized protein YdbJ | ydbJ | OM | 1 (1) | 2 (2) | 0 (0) | 1 (1) | 1 (1) | 0 (0) |
| YDCL_ECOLI | Hypothetical lipoprotein ydcL | ydcL | OM | 17 (11) | 21 (12) | 19 (13) | 24 (12) | 20 (13) | 22 (13) |
| YDDW_ECOLI | UPF0748 lipoprotein YddW | yddW | OM | 11 (10) | 12 (11) | 10 (10) | 12 (12) | 10 (10) | 9 (9) |
| YDGA_ECOLI | Uncharacterized protein | ydgA | p-IM | 20 (20) | 21 (19) | 20 (19) | 30 (28) | 25 (23) | 27 (25) |
| YDGH_ECOLI | Protein ydgH | ydgH | P | 5 (5) | 11 (9) | 4 (4) | 7 (7) | 2 (2) | 7 (7) |
| YDIY_ECOLI | Uncharacterized protein | ydiY | P | 11 (9) | 13 (9) | 10 (7) | 7 (6) | 13 (10) | 4 (4) |
| YDJN_ECOLI | Hypothetical symporter ydjN | ydjN | IM | 6 (5) | 8 (6) | 6 (5) | 11 (7) | 7 (5) | 14 (8) |
| YEAY_ECOLI | Uncharacterized lipoprotein YeaY | yeaY | OM | 9 (9) | 8 (8) | 10 (10) | 9 (8) | 11 (11) | 9 (9) |
| YEBT_ECOLI | Uncharacterized protein | yebT | IM | 0 (0) | 1 (1) | 0 (0) | 5 (5) | 0 (0) | 2 (2) |
| YECR_ECOLI | Uncharacterized protein | yecR | OM | 3 (2) | 4 (2) | 4 (2) | 4 (2) | 4 (2) | 4 (2) |
| YEDD_ECOLI | Hypothetical lipoprotein yedD | yedD | OM | 23 (13) | 27 (13) | 19 (13) | 21 (12) | 20 (13) | 24 (12) |
| YEGH_ECOLI | Uncharacterized protein | yegH | IM | 5 (5) | 2 (2) | 3 (3) | 3 (3) | 1 (1) | 3 (3) |
| YEIU_ECOLI | Uncharacterized protein | yeiU | IM | 1 (1) | 3 (3) | 1 (1) | 3 (3) | 2 (2) | 2 (2) |
| YEJM_ECOLI | Inner membrane protein YejM | yejM | IM | 1 (1) | 2 (2) | 2 (2) | 7 (6) | 1 (1) | 3 (3) |
| YFAZ_ECOLI | Uncharacterized protein | yfaZ | OM | 6 (4) | 6 (4) | 6 (4) | 5 (4) | 7 (5) | 7 (4) |
| YFGM_ECOLI | Uncharacterized protein | yfgM | IM | 4 (4) | 8 (7) | 3 (3) | 8 (7) | 4 (4) | 14 (12) |
| YFHG_ECOLI | Uncharacterized protein | yfhG | OM | 6 (5) | 4 (3) | 5 (3) | 0 (0) | 4 (4) | 2 (2) |
| YFIB_ECOLI | Putative lipoprotein yfiB | yfiB | OM | 1 (1) | 5 (4) | 3 (3) | 3 (2) | 2 (2) | 7 (5) |
| YGAP_ECOLI | Uncharacterized protein | ygaP | IM | 2 (2) | 3 (3) | 1 (1) | 4 (3) | 2 (2) | 3 (2) |
| YGAU_ECOLI | Uncharacterized protein YgaU | ygaU | p-IM | 11 (8) | 11 (7) | 8 (6) | 8 (5) | 10 (8) | 14 (11) |
| YGDR_ECOLI | Uncharacterized lipoprotein YgdR | ygdR | OM | 4 (4) | 6 (4) | 7 (5) | 4 (4) | 3 (3) | 5 (4) |
| YGER_ECOLI | Hypothetical lipoprotein ygeR | ygeR | OM | 16 (14) | 17 (15) | 17 (14) | 7 (6) | 12 (11) | 4 (3) |
| YGGE_ECOLI | Uncharacterized protein | yggE | P | 0 (0) | 2 (2) | 0 (0) | 11 (10) | 0 (0) | 1 (1) |
| YGHB_ECOLI | Inner membrane protein YghB | yghB | IM | 1 (1) | 2 (2) | 1 (1) | 2 (2) | 1 (1) | 1 (1) |
| YGIM_ECOLI | Uncharacterized protein YgiM | ygiM | IM | 2 (2) | 5 (5) | 5 (5) | 7 (7) | 3 (3) | 7 (7) |
| YHCB_ECOLI | Putative cytochrome d ubiquinol oxidase subunit 3 | yhcB | IM | 26 (10) | 27 (11) | 28 (9) | 33 (11) | 23 (9) | 23 (10) |
| YHDP_ECOLI | Uncharacterized protein | yhdP | OM | 2 (2) | 6 (5) | 0 (0) | 2 (2) | 4 (4) | 1 (1) |
| YHES_ECOLI | Hypothetical ABC transporter ATP-binding protein yheS | yheS | p-IM | 1 (1) | 4 (3) | 3 (3) | 1 (1) | 3 (3) | 4 (4) |
| YHFL_ECOLI | Uncharacterized protein YhfL | yhfL | OM | 1 (1) | 2 (2) | 0 (0) | 0 (0) | 0 (0) | 0 (0) |
| YHGF_ECOLI | Protein yhgF | yhgF | p-IM | 4 (4) | 6 (6) | 7 (7) | 4 (4) | 5 (5) | 6 (6) |
| YHHM_ECOLI | Uncharacterized protein | yhhM | IM | 0 (0) | 3 (3) | 0 (0) | 4 (2) | 0 (0) | 2 (2) |
| YHII_ECOLI | Uncharacterized protein | yhiI | IM | 1 (1) | 2 (2) | 2 (2) | 6 (6) | 0 (0) | 7 (7) |
| YHJD_ECOLI | Uncharacterized protein | yhjD | IM | 2 (2) | 3 (2) | 1 (1) | 4 (3) | 2 (2) | 1 (1) |
| YIAD_ECOLI | Hypothetical lipoprotein yiaD | yiaD | IM, IM | 20 (11) | 22 (9) | 15 (9) | 17 (8) | 14 (9) | 19 (9) |
| YIAF_ECOLI | Uncharacterized protein YiaF | yiaF | IM | 6 (5) | 7 (6) | 5 (4) | 8 (7) | 5 (4) | 9 (7) |
| YIBN_ECOLI | Uncharacterized protein YibN | yibN | IM | 19 (8) | 20 (9) | 19 (8) | 19 (9) | 21 (8) | 16 (9) |
| YICH_ECOLI | Uncharacterized protein | yicH | IM | 2 (2) | 2 (2) | 4 (4) | 3 (3) | 5 (5) | 4 (4) |
| YIDC_ECOLI | Membrane protein insertase YidC | yidC | IM | 33 (27) | 42 (24) | 25 (20) | 45 (22) | 37 (25) | 33 (23) |
| YIDQ_ECOLI | Uncharacterized protein | yidQ | IM | 3 (3) | 6 (4) | 3 (2) | 5 (4) | 2 (2) | 3 (3) |
| YIFL_ECOLI | Uncharacterized lipoprotein YifL | yifL | OM | 16 (4) | 14 (4) | 16 (4) | 11 (3) | 12 (3) | 11 (4) |
| YJEI_ECOLI | Uncharacterized protein YjeI | yjeI | OM | 7 (3) | 8 (3) | 7 (3) | 10 (3) | 6 (3) | 6 (3) |
| YJGA_ECOLI | UPF0307 protein YjgA | yjgA | p-IM | 3 (3) | 3 (3) | 1 (1) | 0 (0) | 0 (0) | 0 (0) |
| YJIY_ECOLI | Uncharacterized protein | yjiY | IM | 11 (6) | 17 (9) | 14 (8) | 20 (11) | 10 (5) | 20 (12) |
| YJJK_ECOLI | ABC transporter ATP-binding protein yjjK | yjjK | p-IM | 25 (21) | 15 (14) | 17 (16) | 18 (16) | 24 (20) | 23 (20) |
| YMBA_ECOLI | Uncharacterized protein | ymbA | IM | 6 (6) | 2 (2) | 2 (2) | 3 (3) | 6 (5) | 10 (7) |
| YMGG_ECOLI | UPF0757 protein YmgG | ymgG | IM | 7 (3) | 6 (4) | 8 (4) | 7 (4) | 7 (4) | 7 (3) |
| YNBE_ECOLI | Uncharacterized protein YnbE | ynbE | OM | 6 (5) | 5 (4) | 6 (4) | 4 (3) | 6 (5) | 4 (3) |
| YNFB_ECOLI | UPF0482 protein YnfB | ynfB | OM | 8 (7) | 6 (5) | 8 (7) | 7 (6) | 6 (6) | 6 (5) |
| YNIB_ECOLI | Uncharacterized protein | yniB | IM | 1 (1) | 2 (2) | 1 (1) | 6 (4) | 1 (1) | 1 (1) |
| YOAE_ECOLI | UPF0053 inner membrane protein YoaE | yoaE | IM | 1 (1) | 2 (2) | 2 (2) | 5 (5) | 3 (3) | 2 (2) |
| YOAF_ECOLI | Uncharacterized protein YoaF | yoaF | OM | 2 (2) | 4 (2) | 2 (2) | 2 (1) | 3 (2) | 1 (1) |
| YOJI_ECOLI | Hypothetical ABC transporter ATP-binding protein yojI | yojI | IM | 0 (0) | 3 (3) | 0 (0) | 8 (7) | 1 (1) | 6 (6) |
| YPFN_ECOLI | UPF0370 protein YpfN | ypfN | IM | 1 (1) | 1 (1) | 1 (1) | 1 (1) | 0 (0) | 0 (0) |
| YQEG_ECOLI | Inner membrane transport protein YqeG | yqeG | IM | 1 (1) | 3 (3) | 2 (2) | 4 (3) | 1 (1) | 2 (2) |
| YQHA_ECOLI | UPF0114 protein YqhA | yqhA | IM | 2 (2) | 3 (2) | 1 (1) | 5 (3) | 2 (2) | 2 (1) |
| YRAP_ECOLI | Uncharacterized protein YraP | yraP | OM | 19 (12) | 19 (11) | 22 (13) | 19 (12) | 21 (13) | 19 (14) |
| ZIPA_ECOLI | Cell division protein ZipA homolog | zipA | IM | 3 (3) | 4 (4) | 3 (3) | 5 (5) | 2 (2) | 6 (6) |
|  | Uncharacterized protein | c3694 | - | 111 (71) | 135 (61) | 116 (73) | 172 (72) | 102 (69) | 120 (64) |
|  | Putative iron-regulated outer membrane virulence protein | c5174 | - | 95 (40) | 69 (33) | 82 (45) | 80 (41) | 74 (42) | 49 (26) |
|  | Antigen 43 | c3655 | - | 75 (48) | 52 (39) | 57 (39) | 41 (33) | 74 (49) | 39 (32) |
|  | Outer membrane porin protein nmpC | c2348 | - | 71 (7) | 72 (8) | 63 (7) | 75 (6) | 106 (6) | 63 (8) |
|  | Cellulose synthase operon protein C | yhjL | - | 67 (56) | 57 (48) | 57 (49) | 59 (51) | 58 (48) | 54 (47) |
|  | IutA protein | iutA | - | 57 (41) | 44 (29) | 49 (40) | 60 (30) | 48 (37) | 34 (28) |
|  | F1C fimbrial usher | focD | - | 41 (36) | 26 (24) | 37 (35) | 38 (35) | 43 (38) | 30 (26) |
|  | Hemolysin A | hlyA | - | 34 (31) | 65 (33) | 26 (22) | 34 (25) | 6 (6) | 34 (27) |
|  | MltA-interacting protein | yeaF | - | 30 (14) | 24 (10) | 30 (15) | 23 (11) | 26 (12) | 21 (10) |
|  | Uncharacterized protein | ytfM | - | 30 (23) | 26 (21) | 31 (26) | 29 (25) | 31 (25) | 28 (24) |
|  | Uncharacterized protein | c1204 | - | 29 (9) | 21 (9) | 25 (9) | 24 (9) | 29 (9) | 16 (9) |
|  | PapC protein | papC_2 | - | 25 (23) | 26 (21) | 23 (21) | 31 (25) | 22 (18) | 21 (17) |
|  | Putative receptor | c3610 | - | 25 (15) | 33 (18) | 24 (15) | 22 (19) | 26 (15) | 23 (14) |
|  | Mannose-1-phosphate guanylyltransferase | c2558 | - | 24 (22) | 17 (16) | 19 (18) | 18 (17) | 26 (21) | 15 (14) |
|  | Outer membrane heme/hemoglobin receptor | chuA | - | 23 (21) | 29 (25) | 16 (15) | 21 (20) | 21 (20) | 26 (22) |
|  | Siderophore receptor IroN | iroN | - | 22 (21) | 19 (18) | 18 (18) | 19 (18) | 16 (16) | 12 (12) |
|  | Protein yijP | yijP | - | 20 (19) | 21 (16) | 20 (18) | 19 (19) | 28 (18) | 19 (19) |
|  | Cyclic di-GMP binding protein | yhjN | - | 19 (19) | 25 (22) | 20 (20) | 28 (25) | 21 (20) | 30 (29) |
|  | Uncharacterized protein | c5373 | - | 17 (16) | 11 (11) | 11 (9) | 8 (8) | 12 (11) | 14 (13) |
|  | Protease do | htrA | - | 16 (14) | 19 (17) | 22 (19) | 25 (21) | 21 (18) | 17 (16) |
|  | Uncharacterized protein | ytfN | - | 16 (16) | 19 (17) | 18 (18) | 16 (16) | 16 (15) | 7 (7) |
|  | Potassium efflux system kefA | aefA | - | 16 (16) | 24 (22) | 15 (15) | 27 (26) | 18 (17) | 27 (25) |
|  | Uncharacterized protein | c5372 | - | 15 (13) | 19 (18) | 10 (10) | 23 (22) | 14 (14) | 19 (18) |
|  | Serine protease pic autotransporter | pic | - | 14 (12) | 13 (11) | 11 (9) | 8 (6) | 13 (12) | 6 (5) |
|  | Putative glycerol-3-phosphate cytidyltransferase | c3696 | - | 14 (9) | 13 (7) | 10 (6) | 11 (6) | 9 (5) | 6 (5) |
|  | Serine protease sat autotransporter | sat | - | 14 (12) | 17 (16) | 11 (9) | 9 (8) | 14 (12) | 6 (5) |
|  | VacJ lipoprotein | vacJ | - | 13 (6) | 13 (7) | 10 (5) | 12 (6) | 8 (6) | 10 (6) |
|  | Copper homeostasis protein cutF | cutF | - | 13 (12) | 13 (12) | 20 (11) | 16 (9) | 17 (15) | 11 (10) |
|  | Penicillin-binding protein activator LpoB | ycfM | - | 12 (8) | 11 (8) | 11 (8) | 8 (7) | 18 (8) | 13 (8) |
|  | Lipoprotein | yaeC | - | 12 (6) | 12 (9) | 13 (6) | 10 (6) | 10 (5) | 15 (8) |
|  | UDP-glucose 4-epimerase | c2560 | - | 11 (9) | 6 (6) | 7 (6) | 6 (4) | 7 (6) | 6 (5) |
|  | Uncharacterized protein | yggB | - | 8 (8) | 5 (5) | 5 (5) | 6 (6) | 5 (5) | 7 (6) |
|  | Uncharacterized protein | c2559 | - | 8 (8) | 11 (10) | 9 (9) | 16 (12) | 10 (10) | 12 (10) |
|  | DcrB protein | c4265 | - | 8 (7) | 5 (4) | 7 (6) | 10 (9) | 7 (7) | 15 (13) |
|  | Putative outer membrane receptor for iron compound or colicin | c2482 | - | 7 (6) | 6 (6) | 7 (6) | 5 (4) | 5 (4) | 1 (1) |
|  | Putative iron compound receptor | c3775 | - | 7 (7) | 12 (11) | 5 (5) | 12 (11) | 7 (5) | 9 (9) |
|  | Uncharacterized protein | c4510 | - | 7 (7) | 11 (10) | 8 (7) | 12 (11) | 10 (9) | 8 (8) |
|  | Ribosome-binding ATPase YchF | ychF | - | 7 (7) | 5 (5) | 8 (8) | 5 (5) | 6 (6) | 7 (7) |
|  | Capsule polysaccharide export inner-membrane protein KpsE | kpsE | - | 6 (6) | 10 (10) | 6 (6) | 13 (13) | 8 (8) | 15 (15) |
|  | Uncharacterized protein | c3601 | - | 6 (4) | 2 (2) | 4 (3) | 3 (2) | 4 (3) | 2 (2) |
|  | Uncharacterized protein | c0945 | - | 6 (5) | 5 (3) | 3 (3) | 14 (9) | 6 (6) | 6 (5) |
|  | Partial tonB-like membrane protein encoded within prophage | c1565 | - | 6 (4) | 5 (5) | 7 (5) | 5 (5) | 3 (3) | 4 (4) |
|  | Hypothetical transport protein yeeF | yeeF | - | 5 (5) | 5 (4) | 5 (5) | 5 (3) | 2 (2) | 4 (4) |
|  | Uncharacterized protein | c1190 | - | 5 (5) | 5 (5) | 5 (5) | 6 (5) | 5 (4) | 5 (5) |
|  | Uncharacterized protein | yrbD | - | 5 (4) | 5 (5) | 5 (5) | 5 (5) | 4 (4) | 7 (6) |
|  | Partial Putative outer membrane channel protein | c1765 | - | 5 (5) | 3 (3) | 5 (5) | 1 (1) | 4 (4) | 3 (3) |
|  | Outer membrane protein assembly factor BamE | bamE | OM | 5 (2) | 2 (1) | 6 (2) | 4 (2) | 3 (1) | 4 (1) |
|  | L-serine dehydratase 1 | c3870 | - | 5 (3) | 2 (2) | 4 (3) | 1 (1) | 6 (4) | 4 (3) |
|  | Uncharacterized protein | c3693 | - | 5 (5) | 7 (6) | 2 (2) | 3 (2) | 2 (2) | 1 (1) |
|  | Uncharacterized protein | c3635 | - | 4 (4) | 5 (3) | 4 (4) | 6 (3) | 6 (3) | 3 (3) |
|  | PapA protein | papA_2 | - | 4 (4) | 3 (3) | 3 (3) | 4 (4) | 4 (4) | 3 (3) |
|  | Probable N-acetylmuramoyl-L-alanine amidase ybjR | ybjR | - | 4 (4) | 5 (5) | 4 (4) | 1 (1) | 5 (5) | 1 (1) |
|  | Putative Na(+)/H(+) exchanger yjcE | c5057 |  | 4 (4) | 4 (4) | 5 (4) | 7 (5) | 5 (5) | 8 (5) |
|  | Uncharacterized protein | c5056 | - | 4 (3) | 4 (3) | 4 (3) | 4 (3) | 4 (3) | 4 (3) |
|  | Uncharacterized protein | c3695 | - | 4 (3) | 7 (6) | 4 (4) | 13 (10) | 3 (2) | 9 (8) |
|  | Uncharacterized protein | c3206 | - | 4 (4) | 5 (5) | 2 (2) | 1 (1) | 1 (1) | 2 (2) |
|  | Uncharacterized protein | yjeP | - | 4 (4) | 9 (8) | 1 (1) | 9 (9) | 6 (6) | 7 (7) |
|  | Putative outer membrane protein of prophage | c3153 | - | 4 (3) | 3 (3) | 4 (3) | 2 (2) | 5 (4) | 3 (3) |
|  | Transport permease protein | kpsM | - | 3 (3) | 1 (1) | 2 (2) | 2 (2) | 2 (2) | 2 (2) |
|  | YapH homolog | c2895 | - | 3 (3) | 1 (1) | 1 (1) | 1 (1) | 0 (0) | 0 (0) |
|  | Protease ecfE | yaeL | - | 3 (3) | 5 (5) | 3 (3) | 9 (7) | 4 (4) | 5 (5) |
|  | TonB dependent receptor | c0294 | - | 3 (3) | 0 (0) | 3 (3) | 0 (0) | 2 (2) | 3 (3) |
|  | Bor protein homolog from lambdoid prophage DLP12 | ybcU | - | 3 (1) | 5 (2) | 4 (2) | 2 (1) | 2 (1) | 4 (1) |
|  | Uncharacterized protein | yjdB | - | 3 (3) | 4 (4) | 2 (2) | 6 (6) | 2 (2) | 2 (2) |
|  | Uncharacterized protein | ydhA | - | 3 (3) | 4 (4) | 4 (4) | 3 (3) | 3 (3) | 4 (4) |
|  | Uncharacterized protein | yciS | - | 3 (3) | 7 (4) | 2 (1) | 11 (4) | 4 (2) | 7 (4) |
|  | Chaperone protein FocC | focC | - | 3 (3) | 3 (2) | 2 (2) | 5 (4) | 1 (1) | 4 (4) |
|  | Lipid A-core, surface polymer ligase | waaL | - | 3 (3) | 2 (2) | 3 (3) | 6 (4) | 2 (2) | 2 (2) |
|  | Uncharacterized protein | c0946 | - | 3 (3) | 2 (2) | 4 (4) | 5 (5) | 3 (3) | 2 (2) |
|  | Glycosyl transferase | c2563 | - | 3 (3) | 11 (10) | 12 (10) | 12 (10) | 7 (6) | 12 (11) |
|  | Uncharacterized protein | c4509 | - | 2 (2) | 5 (4) | 1 (1) | 5 (4) | 1 (1) | 4 (4) |
|  | Isocitrate dehydrogenase [NADP] | icdA | - | 2 (2) | 3 (3) | 7 (6) | 3 (3) | 2 (2) | 3 (3) |
|  | Uncharacterized protein | c3205 | - | 2 (2) | 4 (3) | 3 (3) | 0 (0) | 1 (1) | 1 (1) |
|  | Uncharacterized protein | c0651 | - | 2 (2) | 4 (4) | 3 (3) | 5 (5) | 2 (1) | 5 (5) |
|  | Fructose-bisphosphate aldolase class II | fba | - | 2 (2) | 0 (0) | 6 (5) | 1 (1) | 3 (3) | 1 (1) |
|  | Hemolysin D | hlyD | - | 2 (2) | 6 (6) | 6 (5) | 12 (11) | 7 (6) | 7 (7) |
|  | 2-iminobutanoate/2-iminopropanoate deaminase | yjgF | - | 1 (1) | 0 (0) | 4 (3) | 0 (0) | 2 (2) | 0 (0) |
|  | Copper-transporting P-type ATPase | ybaR | - | 1 (1) | 7 (7) | 1 (1) | 8 (8) | 2 (2) | 3 (3) |
|  | DNA recombination protein rmuC | yigN | - | 1 (1) | 4 (4) | 1 (1) | 5 (5) | 0 (0) | 0 (0) |
|  | Putative protease La homolog | c1091 | - | 1 (1) | 4 (3) | 1 (1) | 5 (5) | 2 (2) | 6 (6) |
|  | Lipopolysaccharide assembly protein B | lapB | - | 1 (1) | 0 (0) | 0 (0) | 4 (3) | 0 (0) | 0 (0) |
|  | Uncharacterized protein | yhjU | - | 1 (1) | 0 (0) | 3 (3) | 4 (4) | 0 (0) | 3 (3) |
|  | Uncharacterized protein | c2405 | - | 1 (1) | 5 (5) | 1 (1) | 4 (4) | 1 (1) | 1 (1) |
|  | DNA repair protein radA | sms | - | 1 (1) | 6 (6) | 1 (1) | 4 (4) | 2 (2) | 3 (3) |
|  | Uncharacterized protein | c0467 | - | 1 (1) | 1 (1) | 0 (0) | 2 (2) | 7 (7) | 0 (0) |
|  | Murein DD-endopeptidase MepS/Murein LD-carboxypeptidase | mepS | - | 1 (1) | 1 (1) | 0 (0) | 1 (1) | 1 (1) | 0 (0) |
|  | Uncharacterized protein | yibP | - | 1 (1) | 1 (1) | 0 (0) | 8 (7) | 3 (3) | 3 (3) |
|  | Uncharacterized protein | c3394 | - | 1 (1) | 2 (2) | 3 (3) | 1 (1) | 3 (3) | 0 (0) |
|  | Protein yeeZ | c2543 | - | 1 (1) | 0 (0) | 1 (1) | 0 (0) | 0 (0) | 0 (0) |
|  | Uncharacterized protein | ybdG | - | 1 (1) | 2 (1) | 3 (3) | 7 (7) | 3 (2) | 9 (8) |
|  | NADP-dependent malic enzyme | c2988 | - | 1 (1) | 3 (3) | 4 (4) | 4 (4) | 3 (3) | 0 (0) |
|  | Putative sensor-like histidine kinase yojN | yojN | - | 0 (0) | 7 (7) | 0 (0) | 4 (4) | 0 (0) | 5 (5) |
|  | KpsT protein | kpsT | - | 0 (0) | 4 (4) | 0 (0) | 5 (4) | 1 (1) | 0 (0) |
|  | Uncharacterized protein | c2561 | - | 0 (0) | 4 (3) | 3 (2) | 6 (5) | 0 (0) | 6 (6) |
|  | F1C Putative fimbrial adhesin | focH | - | 0 (0) | 4 (4) | 2 (2) | 5 (5) | 1 (1) | 1 (1) |
|  | Dipeptide and tripeptide permease B | yhiP | - | 0 (0) | 3 (3) | 2 (2) | 1 (1) | 1 (1) | 4 (3) |
|  | FxsA protein | c5223 | - | 0 (0) | 3 (2) | 0 (0) | 5 (3) | 2 (2) | 3 (2) |
|  | Ribonuclease R | vacB | - | 0 (0) | 2 (2) | 2 (2) | 5 (5) | 0 (0) | 4 (4) |
|  | KpsS protein | kpsS | - | 0 (0) | 1 (1) | 0 (0) | 4 (4) | 0 (0) | 0 (0) |

CL ^a^ = cellular localization; OM=outer membrane; IM=inner membrane; p-IM=peripherally localized to the IM; P=periplasm; “-“=no localization annotated in *STEP*db (<http://www.stepdb.eu/step2/>)
