# Supplementary material for: Peptidoglycan Association of Murein Lipoprotein Is Required for KpsD-Dependent Group 2 Capsular Polysaccharide Expression and Serum Resistance in a Uropathogenic Escherichia coli Isolate
Source: mBio. 2017 May 23;8(3):e00603-17. doi: 10.1128/mBio.00603-17 (PMC5442458; doi:10.1128/mBio.00603-17)
Supplement: TABLE S5 [file mbo003173319st5.docx]

**Table S5:** Human proteins associated with WT CFT073 and CFT073*lpp* total membranes identified through LC/MS/MS

| Uniprot | Description | Gene | Total (Unique) Peptides | | | | | |
| --- | --- | --- | --- | --- | --- | --- | --- | --- |
|  |  |  | CFT073  (LB) | *lpp*  (LB) | CFT073  (nHS) | *lpp*  (nHS) | CFT073  (HIHS) | *lpp*  (HIHS) |
| PROP_HUMAN | Properdin | CFP | 0 (0) | 0 (0) | 0 (0) | 6 (6) | 0 (0) | 0 (0) |
| CO3_HUMAN | Complement C3 | C3 | 0 (0) | 0 (0) | 80 (49) | 296 (97) | 7 (7) | 14 (12) |
| CO5_HUMAN | Complement C5 | C5 | 0 (0) | 0 (0) | 0 (0) | 51 (37) | 0 (0) | 0 (0) |
| CO6_HUMAN | Complement component C6 | C6 | 0 (0) | 0 (0) | 0 (0) | 31 (25) | 0 (0) | 0 (0) |
| CO7_HUMAN | Complement component C7 | C7 | 0 (0) | 0 (0) | 0 (0) | 40 (28) | 0 (0) | 0 (0) |
| CO8A_HUMAN | Complement component C8 α chain | C8A | 0 (0) | 0 (0) | 0 (0) | 13 (12) | 0 (0) | 0 (0) |
| CO8B_HUMAN | Complement component C8 β chain | C8B | 0 (0) | 0 (0) | 0 (0) | 13 (12) | 0 (0) | 0 (0) |
| CO8G_HUMAN | Complement component C8 γ chain | C8G | 0 (0) | 0 (0) | 0 (0) | 5 (5) | 0 (0) | 0 (0) |
| CO9_HUMAN | Complement component C9 | C9 | 0 (0) | 0 (0) | 1 (1) | 40 (20) | 0 (0) | 0 (0) |
| IGHM_HUMAN | Ig μ chain C region | IGHM | 0 (0) | 0 (0) | 16 (14) | 25 (14) | 7 (6) | 14 (10) |
| IGKC_HUMAN | Ig κ chain C region | IGKC | 0 (0) | 0 (0) | 13 (6) | 22 (6) | 7 (4) | 10 (5) |
| IGHG2_HUMAN | Ig γ-2 chain C region | IGHG2 | 0 (0) | 0 (0) | 9 (3) | 27 (7) | 4 (3) | 11 (6) |
| IGHG1_HUMAN | Ig γ-1 chain C region | IGHG1 | 0 (0) | 0 (0) | 9 (7) | 19 (11) | 7 (7) | 10 (7) |
| IGHA1_HUMAN | Ig α-1 chain C region | IGHA1 | 0 (0) | 0 (0) | 13 (10) | 16 (12) | 1 (1) | 5 (5) |
| APOB_HUMAN | Apolipoprotein B-100 | APOB | 0 (0) | 0 (0) | 0 (0) | 19 (18) | 3 (3) | 243 (137) |
| FINC_HUMAN | Fibronectin | FN1 | 0 (0) | 0 (0) | 6 (6) | 18 (17) | 1 (1) | 46 (38) |
| FIBB_HUMAN | Fibrinogen β chain | FGB | 0 (0) | 0 (0) | 5 (5) | 8 (8) | 0 (0) | 5 (5) |
