# Supplementary material for: Peptidoglycan Association of Murein Lipoprotein Is Required for KpsD-Dependent Group 2 Capsular Polysaccharide Expression and Serum Resistance in a Uropathogenic Escherichia coli Isolate
Source: mBio. 2017 May 23;8(3):e00603-17. doi: 10.1128/mBio.00603-17 (PMC5442458; doi:10.1128/mBio.00603-17)
Supplement: TABLE S6 [file mbo003173319st6.doc]

**Table S6:** Bacterial strains and plasmids

| **Strain** | **Description/Use** | **Source** |
| --- | --- | --- |
| ***E. coli*** |  |  |
| CFT073 | Bacteremia isolate, wild-type (O6:K2:H1) | [22] |
| TOP10  CFT073*lpp* | pWQ601, general cloning strain  CFT073 Δ*lpp*::kan | Invitrogen  This study |
| CFT073*pal* | CFT073 Δ*pal*::kan | This study |
| CFT073*c3694* | CFT073 Δ*c3694*::kan | This study |
| CFT073*kpsD* | CFT073 Δ*kpsD*::kan | This study |
| **Plasmids** |  |  |
| pKD4 | Kanamycin resistance (Kan^R^) cassette flanked by FRT (FLP recognition target) sites, oriRγ  | [50] |
| pKD46 | Expresses the phage λ Red recombinase, Amp^R^, temperature sensitive, oriRγ  | [50] |
| pCP20 | Thermal induction of FLP recombinase expression, Amp^R^, temperature sensitive  | [51] |
| pUC | pUC19 high copy plasmid, pMB1 origin | NEB |
| pUC-Lpp | pUC19 vector expressing full-length Lpp under its endogenous promoter (+286) | This study |
| pUC-LppΔK | pUC19 vector expressing Lpp variant with C-terminal Lysine deletion under its endogenous promoter (+286) | This study |
| pUC-KpsD | pUC19 vector expressing KpsD under the Lac promoter | This study |
