# Supplementary material for: Peptidoglycan Association of Murein Lipoprotein Is Required for KpsD-Dependent Group 2 Capsular Polysaccharide Expression and Serum Resistance in a Uropathogenic Escherichia coli Isolate
Source: mBio. 2017 May 23;8(3):e00603-17. doi: 10.1128/mBio.00603-17 (PMC5442458; doi:10.1128/mBio.00603-17)
Supplement: TABLE S7 [file mbo003173319st7.doc]

**Table S7:** Primers used to generate CFT073 mutant strains

| **Primer** | **Sequence (5’ to 3’)** |
| --- | --- |
| CFT073*lpp.*F | GTAATACTTGTAACGCTACATGGAGATTAACTCAATCTAGAGGGTATTAATAGTGTAGGCTGGAGCTGCTTC |
| CFT073*lpp.*R | GGCAAAAAAATGGCGCACAATGTGCGCCATTTTTCACTTCACAGGTACTACATATGAATATCCTCCTTAGTTCCTATTC |
| CFT073*pal.*F | GAACCTGTACGTAATGGATTTG |
| CFT073*pal.*R | CCATTGCGTCATCCTGGCGGGATT |
| CFT073*kpsD.*F | TCGGCACCCTGAAACTGTTGCTGGCTGTTATTGAAGATCACCGAGACTAACGCTGTCGCTGAATGAGTTTGTGTAGGCTGGAGCTGCTTC |
| CFT073*kpsD.*R | GGCTTACCCGGCAGGCGCGATGAGCCATAACGAGCCGGGATGACAATAACAGCTTTGCTCATTTCAACTCCCATATGAATATCCTCCTTAGTTCCTATT |
| CFT073*c3694.*F | GCCTTGAGGTCTATATAACTGAATATATTAAATATTAAAAAATAATCATGGGGACATAGTGTGTAGGCTGGAGCTGCTTC |
| CFT073*c3694.*R | GGAGGTATACCTCCTTACAAATCAATCAATGGTTCTTGACGATCATGATATAGATTAATGCTCATATGAATATCCTCCTTAGTTCCTATTC |
