# Supplementary material for: Peptidoglycan Association of Murein Lipoprotein Is Required for KpsD-Dependent Group 2 Capsular Polysaccharide Expression and Serum Resistance in a Uropathogenic Escherichia coli Isolate
Source: mBio. 2017 May 23;8(3):e00603-17. doi: 10.1128/mBio.00603-17 (PMC5442458; doi:10.1128/mBio.00603-17)
Supplement: TEXT S1 [file mbo003173319s1.docx]

**Supplemental Material**

Peptidoglycan association of murein lipoprotein is required for KpsD-dependent group 2 capsular polysaccharide expression and serum resistance in a uropathogenic *Escherichia coli* isolate

Jingyu Diao^a^, Catrien Bouwman^b^, Donghong Yan^c^, Jing Kang^c^, Anand Kumar Katakam^d^, Peter Liu^e^, Homer Pantua^a^, Alexander R. Abbas^f^, Nicholas N. Nickerson^a^, Cary Austin^d^, Mike Reichelt^d^, Wendy Sandoval^e^, Min Xu^c^, Chris Whitfield^b#^ and Sharookh B. Kapadia^a#^

Supplemental Text

Supplemental Figures S1-S3

Supplemental Tables S1-S7

**Supplementary Text**

**Antibody generation**

**Anti-Lpp antibody.** For recombinant protein expression, a DNA fragment encoding Lpp (Ser_22_-Lys_78_) was cloned into a modified pAcGP67A vector downstream of the polyhedron promoter with an N-terminal His_8_-tag.  Recombinant baculovirus was generated using the Baculogold system (BD Biosciences) following standard protocols and *Trichoplusia ni* cells were infected for protein production and harvested 48 hours post-infection. Cells expressing Lpp (Ser_22_-Lys_78_) were resuspended in lysis buffer (50 mM Tris pH 8.0, 150 mM NaCl, 10% glycerol, 1 mM TCEP, 20 mM imidazole) with cOmplete EDTA-free protease inhibitor tablets (Roche) added and stirred for 1 hour at 4^o^C.  Following three passages through an LV1 microfluidizer (Microfluidics), the lysate was centrofuged at 40,000 × g for 1 hour at 4^o^C and supernatant passed through a 0.45 μm filter.  Clarified supernatant was passed over a Ni-NTA resin and washed with 45 column volumes (CVs) of Buffer A + 0.1% Triton X-114, and then subsequently washed with another 50 CVs of Buffer A (20 mM Tris pH 7.5, 300 mM NaCl, 10% glycerol, 20 mM imidazole, 0.5 mM TCEP).  Protein was eluted with Buffer B (20 mM Tris pH 7.5, 300 mM NaCl, 10% glycerol, 250 mM imidazole, 0.5 mM TCEP) and then passed over a Superdex 200 16/60 column in Buffer C (50 mM Tris pH 7.5, 150 mM NaCl, 10% glycerol, 0.5 mM TCEP).  The peak fraction was pooled and diluted with Buffer D (20 mM MES, pH 6.0, 10% glycerol, 0.5 mM TCEP) to a NaCl concentration of 100 mM and loaded onto a HiTrap SP column.  Following a column wash, the protein was eluted with a gradient of Buffer E (20 mM MES, pH 6.0, 1 M NaCl, 10% glycerol, 0.5 mM TCEP).  Finally, the Lpp peak fraction was collected, concentrated, and desalted into Buffer C. Two rabbits were each immunized with 500 μg Lpp (Ser_22_-Lys_78_) in Complete Freund’s Adjuvant (via multiple sub-cutaneous and intra-dermal site injections) followed up by four biweekly boosts with 250 μg Lpp in Incomplete Freund’s Adjuvant, after which production bleeds were obtained and Lpp affinity-purified polyclonal antibody was generated.

**Anti-KpsD antibody.** KpsD protein was injected into rabbits intramuscularly with Freund’s incomplete adjuvant. The antigen was a variant KpsD protein with an N-terminal hexahistidine tag and TEV-cleavage site preceding residues 21-527 of KpsD. Protein was purified from 2L cultures of *E. coli* TOP10 (pWQ601) grown in LB at 37°C. Cultures were grown to OD ~0.4 and KpsD expression was induced from the pBAD promoter (1) using 0.04% arabinose for another 3 hours. Cells were then collected and resuspended in 20 mM sodium phosphate, pH 7.0 containing cOmplete EDTA-free protease inhibitors and the cells were lysed using an EmulsiFlex homogenizer. The lysate was cleared by sequential centrifugation at 5000 × g for 10 minutes and 15000 × g spin 30 minutes and applied to a disposable Ni-NTA Sepharose column. The column was washed stepwise with 20 mM and then 50 mM imidazole in 50 mM sodium phosphate buffer containing, 300 mM NaCl. Protein was eluted in the same buffer containing 250 mM imidazole. Purified KpsD protein was applied to CNBr-activated Sepharose 4B (GE healthcare) according to the manufacturer’s instructions. Four mL of KpsD serum was diluted in 7mL PBS, added to the to KpsD-bound beads and mixed overnight at 4°C. The initial serum was replaced with fresh PBS-diluted serum and mixed for 1 hour at 4°C. Purified antibodies were eluted from the column using 0.1 M glycine, pH 2.2.

**Measurement of complement component deposition by flow cytometry**

Flow cytometry was used to determine the binding or deposition of complement proteins C1q, Factor B, C3, C5 and C9 on the surface of WT or mutant CFT073 in nHS. Incubation in human serum was performed as described above but samples were harvested after 10 minutes to limit the extent of bacterial lysis. Bacteria were collected by centrifugation at 3000 × g for 5 minutes and resuspended in 100 μL of PBS containing 2.0% BSA prior to incubation with anti-human IgG/IgM-FITC (dilution 1:200, Jackson Immuno Research), anti-C3-FITC (dilution 1:400; Thermo Fisher Scientific), anti-C5-FITC (dilution 1:200, USBiological), anti-C7-PE (dilution 1:100, LSBio), or anti-C9neo (dilution 1:100, Santa Cruz Biotechnology, Inc.) at RT for 1 hour. As controls for samples treated with the complement component-depleted sera, recombinant components were purchased from Complement Technology and added back to the depleted sera according to the manufacturers recommendations. Cells were washed 3x with PBS and resuspended in PBS containing 2% paraformaldehyde (PFA). Flow cytometry analysis was carried out using a FACScan (Becton Dickinson).

**Sucrose gradient centrifugation**

Membranes were resuspended in 3.5 mL of 5 mM EDTA pH 8.0, and loaded onto a two-step gradient composed of 0.3 mL 65% sucrose and 1 mL of 25% sucrose in 5 mM EDTA. Centrifugation was performed for 3 hours at 117,000 × g at 4°C in a swinging bucket rotor and the lower 700 μL, containing the membranes, was collected. This membrane fraction was mixed with 1.3 mL 5 mM EDTA pH 8.0 and loaded onto a sucrose step gradient comprised of 0.5 mL 65% sucrose, 1 mL 55% sucrose, 2 mL 50% sucrose, and 2 mL each of 45%, 40%, and 35% sucrose. All sucrose solutions were prepared in 5 mM EDTA. Samples were centrifuged for 16 hours at 221,000 × g in a swinging bucket rotor at 4°C and 400 μL fractions were collected. IM-free OM fractions from the sucrose gradients were pooled and centrifuged at 100,000 × g to remove most of the sucrose, resuspended in 1 mL 5 mM EDTA, pH 8.0

**References**

1. **Guzman LM**, **Belin D**, **Carson MJ**, **Beckwith J**. 1995. Tight regulation, modulation, and high-level expression by vectors containing the arabinose PBAD promoter. J Bacteriol **177**:4121–4130.
